# Tailoring Renewable Photopolymers with Lignin: Printability, Surface and Antioxidant Properties

**DOI:** 10.1021/acsapm.6c00559

**Published:** 2026-04-24

**Authors:** Marius Bodor, Aurora Lasagabáster-Latorre, Pablo Ligero, Sandra María García-Garabal, María Luisa Sánchez Simón, Sonia Dopico-García, María-José Abad

**Affiliations:** † Campus Industrial de Ferrol, CITENI-Grupo de Polímeros, 16737 Universidade da Coruña, Ferrol, Ferrol 15403, Spain; ‡ Dpto Química Orgánica I, Facultad de Óptica y Optometría, 16734 Universidad Complutense de Madrid, Madrid 28037, Spain; § Facultade de Ciencias, BBP Group, Universidade da Coruña, A Coruña 15008, Spain; ∥ Escuela Politécnica de Ingeniería de Ferrol, Campus Industrial de Ferrol, Universidade da Coruña, Ferrol 15403, Spain

**Keywords:** antioxidant activity, biobased polymers, lignin, photopolymers, 3D printing

## Abstract

This study explores the incorporation of organosolv (LGO) and deep eutectic solvent-derived (LGD) lignin, both native and acrylated, into a 50:50 wt % mixture of two biobased monomers, polyethylene glycol dimethacrylate (PEG200DMA) and 1,10-decanediol diacrylate (C10DA), for digital light processing (DLP). Lignin physicochemical properties, dispersion stability, and UV absorptivity were characterized to predict effects on printability. Native lignin strongly reduced light penetration, requiring higher photoinitiator content and longer exposure times to ensure interlayer adhesion, whereas acrylation partially mitigated this effect via lower absorptivity and reactive acrylate groups. Dynamic mechanical and thermal analyses revealed reduced cross-link density and lower glass transition temperatures for composites containing native lignin, whereas composites with acrylated lignin exhibited higher cross-link density and enhanced thermal stability. All lignin composites exhibited decreases in tensile strength and elongation at break, but native lignin composites showed partial recovery of tensile toughness at moderate loadings along with increased hardness, whereas acrylated lignin composites became more brittle due to increased network stiffness and lignin aggregation. Surface analyses demonstrated smoother finishes, improved resolution, and more homogeneous wettability with native lignin. Antioxidant activity was preserved after printing in both types of native lignin but suppressed after acrylation. Above all, unmodified LGO at 2–4 wt % provided the best balance between smaller particle size, compatibility with the resin used and printing parameters offering the best approach for high-resolution, mechanically robust, and multifunctional biobased DLP resins. The choice of lignin type in sustainable additive manufacturing is critical, depending on the careful selection of lignin-matrix pair, desired final properties and cost-effectiveness.

## Graphical abstract

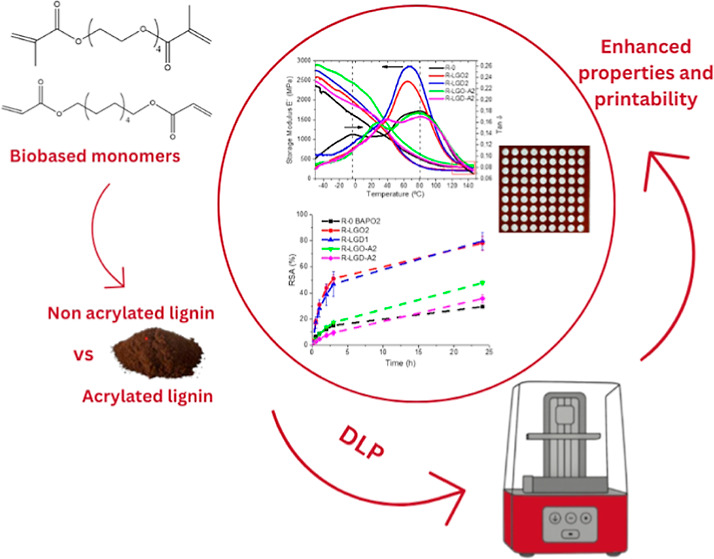

## 1 Introduction

Global interest in sustainability is driving research into biobased alternatives to replace petrochemical-derived resins for 3D printing.^1^ Vat photopolymerization, through stereolithography (SLA) and digital light processing (DLP), stands out for its excellent surface finish and extremely high feature resolution. Recent efforts have been devoted to developing UV-curable liquid epoxy- or (meth)­acrylate-based resins derived from biomass^1^ combining multiple acrylate monomers^2^ or acrylated epoxidized soybean oil (AESO).^3^ To ensure the sustainability of these materials, all additional components should also be sourced from renewable or biobased origins.

Lignin is an abundant phenolic macromolecule and underused industrial byproduct that could play a key role in the development of biobased products. Its structure and final properties highly depend on both its natural origin and the extraction and purification processes, such as the well-known industrial organosolv, soda, or Kraft processes^4^ or more recent extraction methods that use ionic liquids (ILs)^5^ and deep eutectic solvents (DESs).^6^

Lignin can enhance mechanical and thermal properties of both thermoplastic polymers and thermosetting polymer systems.^7^ In additive manufacturing, the commercial use of lignin is still in its early stages, particularly in photocurable resins. However, various applications have already been explored in medicine, food industry, electronics and construction.^6,8,9^ Incorporating native lignin into vat photopolymerization resins presents several challenges.^10^ First, lignin’s UV absorption competes with photoinitiators, reducing curing rate;^11^ higher lignin contents require adjustments in exposure time and layer thickness.^12^ Second, untreated lignin particles tend to be relatively large and structurally complex, causing phase separation and sedimentation during printing.^13^

Thus far, unmodified lignin has been shown to enhance the mechanical properties of photopolymer resins for 3D printing at loadings up to 1 wt %,^14,15^ whereas higher concentrations cause irregular aggregate formation disrupting curing and reducing part quality.^11^ Two main strategies have emerged to address these issues: the use of lignin nanoparticles (LNPs)^13,16^ and chemical modification of of lignin’s multiple reactive groups (hydroxyl, carbonyl, carboxyl) to enhance its solubility, compatibility and/or reactivity with photocurable resins. Common chemical approaches comprise graft copolymerization and, most commonly, hydroxyl acylation through (meth)­acrylate synthesis.^8^ Notable results include incorporation of up to 15 wt % of methacrylated organosolv lignin in SLA resins,^12^ as well as combination of reduction and acylation to improve cure properties and mechanical performance.^17,18^ A similar strategy was used by Böcherer et al., who achieved incorporation of up to ∼40 wt % lignin in DLP biocomposites after decolorization and UV irradiation to reduce lignin’s UV absorbance.^19^ Despite this progress, many studies still report poor compatibility of methacrylated lignin with polymer matrices. Reinforcing effects are generally limited to low contents of (meth)­acrylated-lignin modified by acylation^20^ and processed under high sonication times.^21,22^

Beyond mechanical enhancement, lignin offers additional functions in photocurable resins: it can improve printability and biodegradability,^23^ regulate cure depth via UV absorption,^13^ and act as a photoinitiator^24^ as well as a compatibilizer for conductive fillers.^11,15^ Lignin-based composites enable applications such as printed electronics,^25^ where added lignin can impart antioxidant and antimicrobial properties reducing oxidative stress, and biofouling to extend service life.^4,22,26^ Last but not least, modified lignin with intrinsic shape-memory or self-healing capabilities shows promise for future 4D printing technologies.^6,21^

Within this context, this work examines the effect of lignin incorporation into a newly formulated 50:50 wt % mixture of two partially biobased monomers, polyethylene glycol dimethacrylate (PEG200DMA) and 1,10-decanediol diacrylate (C10DA), designed for DLP printing. This hydrophilic methacrylate–hydrophobic acrylate pairing enables tunable lignin compatibility through polarity effects, while mechanical properties are modulated by combining a rigid dimethacrylate with a more flexible acrylate. The renewable carbon content of both monomers arises from well-established industrial bioderivation routes. In PEG200DMA, the biobased fraction originates from the PEG backbone, which may be produced from ethylene oxide derived from renewable ethylene obtained via bioethanol dehydration.^27,28^ In the case of C10DA, the biobased character stems from the 1,10-decanediol backbone, industrially produced through alkaline cleavage of castor oil-derived ricinoleic acid to sebacic acid, followed by reduction.^29^ In both monomers, esterification with petrochemically derived (meth)­acrylate functionalities reduces the overall renewable carbon content, resulting in biobased renewable carbon (BRC %) values of approximately 50% for PEG200DMA and 60% for C10DA. This monomer combination provides a realistic and scalable formulation for assessing lignin-based strategies aimed at improving the performance of UV-curable resins in additive manufacturing.

Against this background, several variables were investigated, namely lignin type, particle size, UV absorption, compatibility with the matrix and the effect of chemical functionalization. The lignin types included commercial Kraft lignin (LGK) and lignin extracted in our laboratory by organosolv fractionation (LGO) and deep eutectic solvent (DES) extraction (LGD). LGO and LGD were further chemically modified by hydroxyalkylation (LGOH, LGDH) and acrylation (LGO-A, LGD-A). The printed samples were evaluated in terms of spectroscopic, thermal, mechanical, dynamo-mechanical properties, wettability, as well as the ability of lignin to enhance resin printability. As a final point, their antioxidant properties were assessed. In light of the results obtained, the suitability of these two approaches, nonchemical modification and chemical functionalization, is discussed from various perspectives including cost-effectiveness and the selective enhancement of specific material properties.

## 2 Experimental Section

### 2.1 Materials and Samples Preparation

#### 2.1.1 Materials

Polyethylene glycol dimethacrylate (*M*_w_ = 198.22 g/mol, biorenewable carbon content (BRC) = 50%) (M_1_ = PEG200DMA, SARBIO 6201) and 1,10-decanediol diacrylate (C10DA, *M*_w_ = 310.4 g/mol, BRC = 60%) (M_2_ = C10DA, SARBIO 5201) were kindly donated by Sartomer (Arkema, France). Phenylbis (2,4,6-trimethylbenzoyl) phosphine oxide (BAPO, *M*_w_ = 418.46 g/mol) was obtained from Acros (Geel, Belgium). Ethanol, 2-propanol, THF, acetic acid, hydrochloric acid, sulfuric acid, choline chloride-lactic acid, ethylene carbonate, sodium carbonate, and gallic acid were purchased from Scharlau (Sentmenat, Spain). All chemicals were used without further purification. Water was purified with an Elix 3 system (Millipore, Molsheim, France).

#### 2.1.2 Preparation of Lignin Fillers

Kraft lignin (LGK, UPM BioPiva 395, *M*_w_ = 6000 g·mol^–1^, solid content of 95 wt % at 105 °C) was supplied by UPM Biochemicals (Helsinki, Finland).

Lignin was extracted from *Betula alba* wood from the Atlantic Forest (northwest of Spain) by two ways: an optimized acetosolv delignification treatment and, an optimized deep eutectic solvent process.

Acetosolv lignin (LGO) was obtained via an acid-catalyzed acetic acid process. The raw material was mixed with acetic acid and hydrochloric acid at optimized operation conditions,^25^ following 90% acetic acid, 0.2% hydrochloric acid for 55 min at atmospheric boiling temperature. After extraction, rich-cellulose solid and rich-lignin liquor were separated by filtration. Lignin was recovered from the liquor by water-induced precipitation following pH adjustment, washed five times with water, and freeze-dried prior to use. The acetosolv process achieved a delignification efficiency of 82.3%.

DES lignin (LGD) was extracted using a choline chloride–lactic acid mixture designed to maximize recovery of minimally altered lignin. Treatment was performed under optimized conditions at a solid-to-DES ratio of 3%, 120 °C, for 4 h. Solubilized lignin was recovered by water precipitation (water/liquor ratio 3:1) and freeze-dried prior to use. Under these conditions, the DES process reached a delignification efficiency of 87.8% on a dry basis.

LGO and LGD were chemically modified to introduce reactive acrylate groups, through a two-step reaction process as depicted in Scheme 1. For enhancing reactivity of both lignin types, phenolic hydroxyl groups were converted to aliphatic hydroxyl by alkylation with Ethylene Carbonate as is described by Hua et al.^26^ Prior to derivatization, aromatic and aliphatic hydroxyl contents were determined. Based on the hydroxyl group analysis, several assays were conducted to optimize the molar ratio of ethylene carbonate to aromatic hydroxyl groups. Thus, 10 g of dried lignin were introduced into a 250 mL round-bottom flask with selected amount of ethylene carbonate and sodium carbonate, as catalyst. The optimal molar ratios were 1:28 and 1:45 for acetosolv lignin and DES lignin, respectively. The reaction proceeded at 120 °C for 180 min hours under N_2_ atmosphere in a shaking oil bath. The hydroxyethylated derivatives (LGOH and LGDH) were separated by centrifugation at 4000 rpm for 15 min, washed five times with distilled water and freeze-dried.

**Scheme 1:**
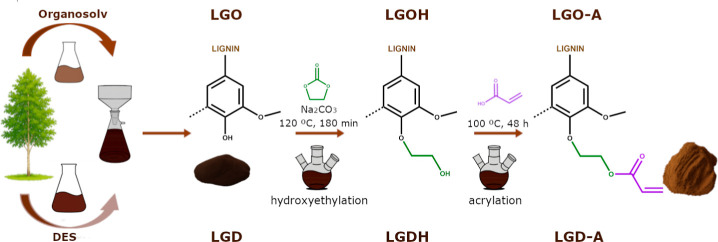
Lignin Extraction from *Betula alba* Wood by Organosolv and Deep Eutectic Solvent (DES) Methods Yielding LGO and LGD, Followed by Hydroxyethylation (LGOH, LGDH) and Acrylation (LGO-A, LGD-A)

The LGOH and LGDH samples were subsequently esterified to yield the corresponding acrylated derivatives, denoted LGO-A and LGD-A. Initially, various lignin/acrylic acid ratios were evaluated, and an optimal ratio of 2 mL of acrylic acid per gram of lignin was identified. This ratio was used to determine the optimal reaction conditions. Reactions were conducted at two temperatures (70 and 100 °C) and three reaction times (24, 48, and 72 h). In all cases, the reaction mixtures were placed in a capped 25 mL round-bottom flask and purged with nitrogen for 5 min prior to heating. The results indicated that reaction times longer than 48 h and temperatures below 100 °C did not improve the reaction efficiency. Accordingly, the hydroxyethylated lignins were acrylated under the optimized conditions by heating the lignin–acrylic acid mixture at 100 °C for 48 h in an oil bath under magnetic stirring. After cooling to room temperature, the reaction mixture was slowly poured into 500 mL of ultrapure water and stirred at 500 rpm for 2 h to remove unreacted acrylic acid. The resulting suspension was centrifuged for 10 min, washed four additional times with ultrapure water, and the recovered solid was freeze-dried.

#### 2.1.3 Preparation of Lignin-Containing 3D-Printable Formulations

Lignin-containing formulations were prepared as described in Table 1, with lignin contents ranging from 1–4 wt % depending on solubility. Samples were compared against the corresponding homopolymers p-M_1_ and p-M_2_, and the neat acrylic resin (R-0). The photoinitiator concentration was adjusted based on UV absorbance (Section 3.2.3), and the biorenewable carbon (BRC %) was calculated^3^ as described in the Supporting Information. Lignin and photoinitiator were added sequentially, each addition followed by 1 min of vortex mixing and 5 min of ultrasonication at 15% amplitude (Digital Sonifier Branson 450, Danbury, CT, USA). In the case of lignin types with larger particle size or lower compatibility with the resin, the mixing protocol was intensified by applying repeated vortex and ultrasonication cycles to ensure proper dispersion. All mixtures were kept in an ice bath throughout sonication to prevent premature polymerization, protected from light, and vortex-mixed for 1 min immediately prior to printing. The chemical formulas of the acrylic monomers and the photoinitiator used to prepare the precursor solutions, as well as a diagram of the preparation process for 3D printing, are shown in Scheme 2.

**Table 1:** Sample Naming, Chemical Composition, Biorenewable Carbon Content (BRC %) and Printing Conditions

formulation	*M*_1_ (%)	*M*_2_ (%)	Lignin (%)	BAPO (%)	BRC (%)	viscosity (mPa·s) at 10 s^–1^	Penetration depth—*D*_p_ (μm)	Transition energy—*E*_c_ (mJ/cm^2^)	exposure time/layer (s)
p-M_1_	100	-	-	1	49.5	8.2	545	31	4
p-M_2_	-	100	-	1	59.4	3.4	398	10	3
R-0	50	50	-	1	54.5	5.6	503	24	4
R-LGO1	50	50	1	1	54.9	11	177	36	10
R-LGO2	50	50	2	2	54.8	11	58	19	20
R-LGO3	50	50	3	3	54.7	15	35	16	50
R-LGO4	50	50	4	4	54.6	17	23	9	101
R-LGD1	50	50	1	3	53.8	11	58	24	20
R-LGD2	50	50	2	6	52.8	19	34	22	60
R-LGO-A2	50	50	2	2	54.8	8.1	81	16	9
R-LGD-A2	50	50	2	2	54.8	13	91	11	6

**Scheme 2:**
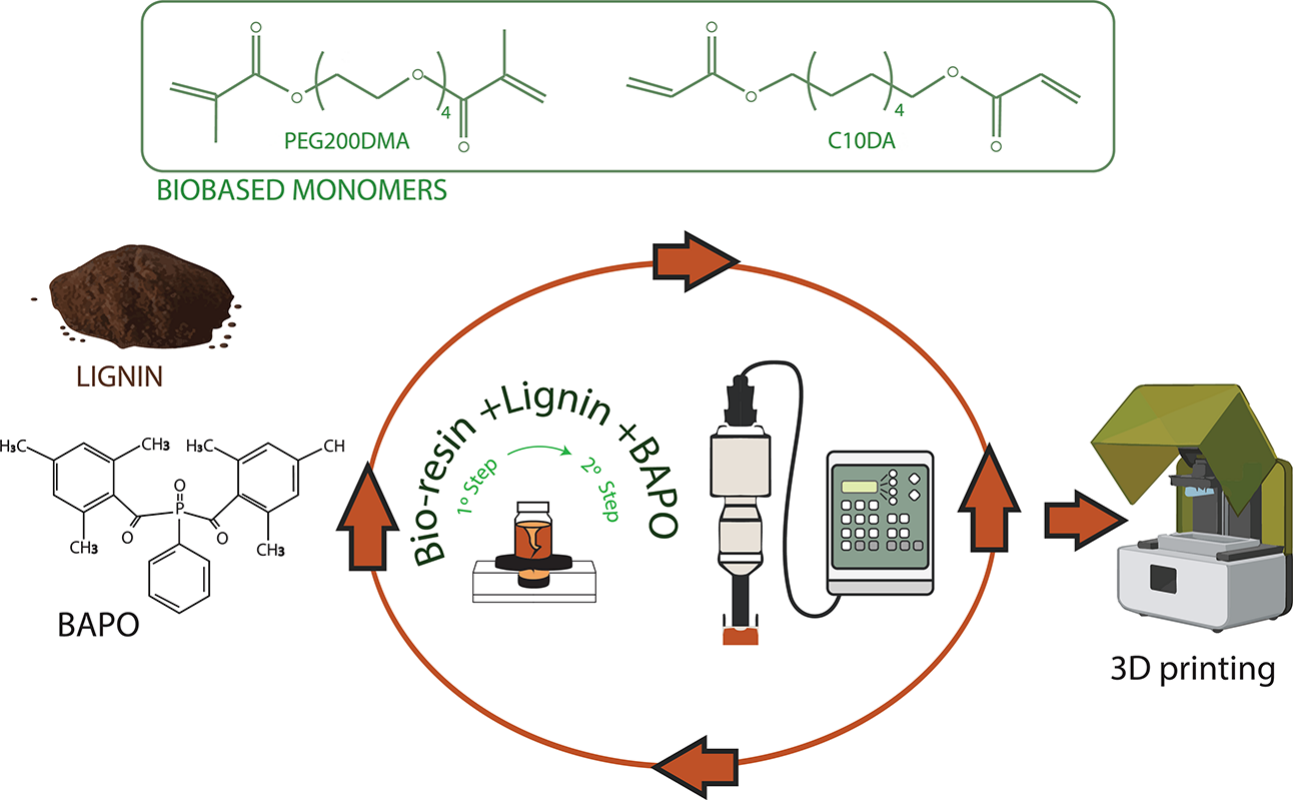
Chemical Structures and Preparation of Lignin-Containing 3D-Printable Formulations

#### 2.1.4 DLP 3D Printing

3D modeling was performed using Chitubox v 1.9.0. The DLP printing was conducted using a modified ELEGOO Mars 3 printer (wavelength 405 nm; 7 mW/cm^2^). Rectangular and dog bone samples were printed horizontally for DMA and tensile testing, respectively. The layer thickness on the *z*-axis was 25 μm, and the exposure time per layer was calculated based on results from UV–vis spectrometry (Section 3.2.3) and the cure depth tests (Section 3.2.4) for an overcure ratio (OR %) of 300% (Table 1). OR is defined as the ratio of Cd to z. OR 300% means that to achieve a 25 μm layer, the theoretical energy corresponding to a 100 μm layer was applied.^30^ This approach was chosen after several trials demonstrated that such overcuring improved both the degree of conversion and the resolution of printed parts. After printing, the samples were soaked in 2-propanol for 10 min to remove uncured monomers, followed by a postcuring process of 5 min at 35 °C, using an incubator (Form Cure, Formlabs, Somerville, MA, USA).

To further assess the printing accuracy and surface finish quality, two representative square plate models with a complex porous structure were selected: (1) a square plate (19.11 × 19.11 × 2.00 mm) with square orifices, each with 1 mm sides, and (2) a square plate (20.02 × 20.02 × 2.00 mm) with circular orifices, each with a diameter of 1.40 mm. Three replicas were printed as described above.

### 2.2 Lignin and Composites Characterization

The particle size distribution of the lignin was measured in aqueous suspension using a laser particle size analyzer (Saturn DigiSizer II 5205, Micromeritics). UV–Vis spectra were recorded on a Jasco V-750 double-beam UV–Vis spectrophotometer (Jasco Analítica S.L., Madrid, Spain) over the 300–800 nm range, with a sampling interval of 1 nm and 25 accumulations.

NMR experiments were performed on a Bruker AVANCE 500 spectrometer equipped with a dual ^1^H/^13^C cryoprobe. The aromatic and aliphatic hydroxyl groups content in lignin was determined by ^31^P NMR spectroscopy as was related by Argyropoulos et al.^31^ Freeze-dried lignin samples were dried overnight in a vacuum oven set at 40 °C. After drying, the lignin was derivatized with 2-chloro-4,4,5,5-tetramethyl-1,3–2-dioxaphospholane (TMDP) in a pyridine/deuterated chloroform mixture, with chromium­(III) acetylacetonate as a relaxation agent and cholesterol as internal standard. The degree of substitution (DS) of aliphatic hydroxyl groups in acrylated lignin was determined by quantitative ^13^C NMR spectroscopy following the protocol described by Hua et al.^26^ The total phenolic content was further estimated colorimetrically using the Folin-Ciocalteu method adapted from Singleton et al.^32^

The viscosity of the liquid monomer formulations was measured at room temperature over a shear-rate range of 0.3–100 s^–1^ using a controlled-strain rheometer (ARES, TA Instruments, Newcastle, DE, USA) equipped with a parallel-plate geometry (25 mm diameter, 1 mm gap). Resin cure properties were determined using the Jacobs working curve model.^33^ The experimental protocol is detailed in a previous study.^34^

Fourier Transformed Infrared (FTIR) spectra were recorded on a Jasco 4700 spectrometer in the attenuated reflectance mode (MIRacle ZnSe Single-Reflection Horizontal ATR accessory), between 4000–550 cm^–1^ at a resolution of 4 cm^–1^ with 64 scans. The yield of the composites’ insoluble fraction (YIF %) was calculated as the ratio of the sample weight after a 16 day acetone extraction to the initial sample weight.^35^

Thermogravimetric Analysis (TGA) was conducted on a TGA 4000—Perkin–Elmer instrument under nitrogen atmosphere with a flow rate of 50 mL min^–1^. For the lignin samples, the temperature was held for 1 min at 35 °C and then increased to 900 °C at a rate of 20.0 ± 0.1 °C·min^–1^. The cured films were subjected to a heating rate of 10.0 ± 0.1 °C min^–1^ from 50 to 700 °C.

DMA tests were performed in a Perkin–Elmer DMA-7 instrument in the three-point bending mode to determine the modulus, the glass transition temperature (*T*_g_) and cross-linking density of the biobased composites. Rectangular printed samples 18 × 6 × 2 mm were tested. The temperature sweep was carried out from −50 to 150 °C at a heating rate of 5 °C·min^–1^ min under nitrogen flow with a deformation frequency of 1 Hz. The storage modulus (É) and damping factor (tan δ) were recorded as a function of the temperature. For each type of resin, three replicate specimens were tested.

Tensile stress–strain properties were tested in an Instron 5569 universal testing machine (Instron Canton, Norwood, MA, USA) with a load cell capacity of 1 kN, at room temperature and crosshead speed of 2 mm·min^–1^ until failure. A minimum of five dog-bone-shaped specimens for each sample were analyzed according to ISO 527:2019.^36^ The hardness of the composites was measured with a Shore D Durometer (Durotech M202, Hampden Test Equipment Limited, Kettering, UK) according to ISO 868:2003.^37^ Readings were taken after 15 s at 10 measuring points per sample.

Scanning electron microscopy (SEM) analyses were performed on a JEOL JSM-7200F microscope at an accelerating voltage of 10 kV to evaluate the fractured side of selected specimens after tensile testing. Prior to observation, the samples were sputter-coated with a thin palladium platinum layer (Cressinton 208HR).

The equilibrium water uptake (*S*_W_) was determined as stated by ISO 62:2008.^38^ Five specimens of each formulation were submerged in water for 24 h, 48 h, 120 h, 288, 480 h (20 days) and 816 h (34 days) and water absorption was measured after wiping the water off. After 34 days of water immersion, the specimens were dried in an oven at 60 °C for 24 h to calculate the hydrolytic degradation (*D*_W_).^34^

Confocal microscopy was used to determine the surface roughness of the printed parts using a PLu 2300 Sensofar optical imaging profiler. Images were captured with an EPI 20× N objective, a depth resolution of 2 μm and a lateral resolution of 1 nm. Areal roughness parameters were obtained using SensoMaP 5.0.4 software, after correction with an F-operator filter, which suppressed the form of the primary surface according to UNE-EN ISO25178-2:2021.^39^ The reported value corresponds to the average of at least three measurements. Surface wettability was assessed by measuring the static contact angle using a Krüss Drop Shape Analyzer DSA25 equipped with an automatic dosing system and a Guppy PRO F-032B CCD camera. Analyses were performed in triplicate using 4 μL deionized water droplets following the sessile drop method outlined in Standard EN 828:2013.^40^ Left and right contact points were averaged, and the final static contact angle was calculated after 10 s. Both the contact angle and surface roughness measurements were performed on the smooth side of horizontally printed samples, corresponding to the last printed layer, as the high roughness of the first layer induced by the metallic platform masked the effect of lignin.

To assess the printing accuracy, the dimensions of the complex porous-structured square plates were first measured with a digital Vernier calliper. Then, two types of images were acquired: (a) pictures taken with a setup consisting of a light source and a cell phone camera with 3× magnification, and (b) pictures captured with the camera of the confocal microscope already described, using a 2.5× N objective. In the case of the square plates with round holes, the latter images were used to evaluate the circularity of the orifices. Eight orifices were measured for each sample using ImageJ software. Circularity was determined using eq 1

1 $$circularity=4\pi \times \frac{\left[area\right]}{{\left[perimeter\right]}^{2}}$$

A value of 1 indicates a perfect circle, while a value approaching 0 indicates an increasingly elongated shape.

The antioxidant capacity of the lignin composites was evaluated at room temperature by assessing their free radical scavenging activity using the DPPH (2,2-diphenyl-1-picrylhydrazyl) spectrophotometric assay, based on the Blois methodology.^41^ Rectangular samples (1.1 ± 0.1 cm^2^, 0.25 ± 0.03 g) were cut from the 3D-printed tensile bars and incubated in amber flasks with 3 mL of the 0.06 mM DPPH solution. The absorbance of the solutions and a control sample were measured at 515 nm at 30 min, 1, 2, 3, and 24 h. The percentage of radical scavenging activity (RSA) was calculated using eq 2

2 $$RSA\left(\%\right)=\frac{\left\lfloor A_{control}-A_{sample}\right\rfloor }{A_{control}}\times 100$$

where *A*_control_ corresponds to the absorbance of the control sample and *A*_sample_ to the neat acrylic resin and lignin composites. RSA values were normalized relative to the area of the samples. All experiments were carried out in triplicate and the results averaged.^42^

For the statistical analysis, data were presented as mean ± standard deviation (SD) and analyzed using Microsoft Excel (Microsoft 365 MSO v. 2401, Redmond, Washington, USA). Differences between means were assessed using Student’s *t*-test or one-way ANOVA and were considered statistically significant at *p* < 0.05.

## 3 Results and Discussion

### 3.1 Characterization of Lignin

The chemical modifications of acetosolv- and DES- lignins were confirmed by combined ^31^P and ^13^C NMR analyses. Quantitative ^31^P NMR revealed a substantial increase in aliphatic hydroxyl groups after hydroxyethylation, accompanied by a corresponding decrease in aromatic hydroxyl groups; this effect was more pronounced for DES lignin than for acetosolv lignin. Following acrylation, the ^31^P NMR spectra showed a strong reduction in aliphatic hydroxyl signals, with conversions of 83% and 84% for acetosolv and DES lignins, respectively (Figures S1 and S2, Table 2). Complementary ^13^C NMR spectra confirmed the formation of ester linkages and the successful incorporation of vinyl functionalities, as evidenced by the appearance of characteristic ester carbonyl and olefinic carbon signals (Figures S3 and S4). The degree of substitution (DS), determined from semiquantitative ^13^C NMR analysis, was 21.3% and 18% for LGO-A and LGD-A, respectively. Minor side reactions leading to carboxylic acid functionalities were detected, particularly for the DES-derived lignin. A detailed spectral analysis, peak assignments, and calculation procedure are provided in the Supporting Information.

**Table 2:** Particle Diameters of Lignin (*D̅*, Mean Diameter; *D*_90_, Percentile Value), Total Phenolic Content Determined by Colorimetry, Phenolic and Aliphatic Hydroxyl Group Contents Quantified by ^31^P NMR, Syringyl (S) and Guaiacyl (G) Ratio (S/G) and UV–Vis Absorptivity Values Measured in THF or THF/H_2_O (1:1, v/v)

	*D̅* (μm)	*D*_90_ (μm)	total phenolics (mg/g)	phenolics ^31^P NMR (mmol/g)	OH aliphatic ^31^P NMR (mmol/g)	OH aliphatic/OH aromatic ratio	S/G ratio FTIR (NMR)	Absorptivity (g/L) 405 nm
LGK	21	69	221 ± 2	2.9	1.9	0.66	0.32	1.25 ± 0.01
LGO	8.7	26	234 ± 4	2.5	1.1	0.45	0.82 (0.63)	1.90 ± 0.20
LGD	75	123	265 ± 4	1.5	0.7	0.48	1.06 (1.61)	5.17 ± 0.11
LGOH	34	87	77 ± 3	0.85	3.0	3.6	0.76	2.30 ± 0.10
LGDH	68	146	66 ± 2	<0.03	1.6	6.7	0.83	5.23 ± 0.24
LGO-A	28	69	-	0.14	<0.03		0.69	-
LGD-A	26	69	-	<0.03	0.25		0.65	-

Additionally, unmodified lignins (LGK, LGO, LGD) and their hydroxyethylated and acrylated derivatives (LGOH, LGDH, LGO-A, LGD-A) were characterized in terms of particle size, phenolic content, UV absorptivity (Table 2) and infrared spectroscopy.

All lignin extracts exhibited a heterogeneous particle size distribution, with notable differences in average particle diameter (Table 2 and Figures S5 and S6). LGO showed the smallest diameter and the highest proportion of fine particles, followed by LGK, while LGD exhibited considerably larger particles. Hydroxyalkylation notably increased the particle size of LGO, whereas it slightly decreased the size of LGD. However, the particle sizes of both acrylated derivatives were similar and intermediate between those of the original lignin.

Free phenolic and aliphatic hydroxyl groups are important features that influence the solubility and reactivity of lignin and its compatibility with acrylic monomers. As shown in Table 2, unmodified lignins are rich in both types of groups: LGK exhibited the highest content of both types of hydroxyl groups, followed by LGO and LGD. As expected, hydroxyalkylation of lignin led to a significant redistribution of hydroxyl groups, whereas subsequent acrylation drastically reduced both types.

FTIR spectra of LGK, LGO and LGD are plotted in Figure S7, with lignin bands assigned according to the literature.^43^ The fingerprint regions of LGO and LGD displayed similar profiles, consistent with their shared origin, whereas significant variations were observed relative to LGK. It is worth mentioning the differences in the bands assigned to syringyl (S) and guaiacyl (G) units. The band at 1325 cm^–1^ corresponds to asymmetric C–O–C stretching vibration in the syringyl ring (S), while the band at 1265 cm^–1^ arises from C–O–C asymmetric stretching in the guaiacyl ring (G).^43^ LGK exhibited the lowest S/G ratio, which is typical of softwood lignin. Although LGD and LGO share the same origin, LGD showed a higher S/G ratio than LGO (Table 2). NMR analysis revealed a similar trend in S/G ratios for these lignins. The lower syringyl content in LGO is likely due to the more acidic conditions of the acetosolv treatment, which promote preferential degradation of syringyl units. For similar reasons, acrylated lignins (LGO-A and LGD-A) displayed lower S/G ratios than their native counterparts.

The efficiency of hydroxyalkylation and acrylation reactions was also confirmed by comparing the spectra native LGO and LGD with those of LGOH and LGDH (Figure S8) and LGO-A and LGD-A (Figure S9), respectively. In particular, the spectra of LGO-A and LGD-A exhibit a significant reduction in the band corresponding to OH groups at 3400 cm^–1^ (ν_OH_), together with a marked increase in the bands assigned to acrylate ester groups at 1770 cm^–1^ (ν_C=O_), 1184 cm^–1^ (ν_C–O–C_) and the in-plane bending vibrations of the =CH2 groups (808 cm^–1^).^12^ These spectral changes confirmed successful acrylation.

### 3.2 Characterization of Lignin/Acrylate UV-Curable Resins

#### 3.2.1 Dispersion Stability of Lignin in Acrylic Resin

UV-curable resins for 3D printing must remain stable and homogeneous throughout the process. To assess this, sedimentation tests were performed on 2 wt % lignin dispersions. Both LGO and LGD exhibited excellent dispersion stability with no phase separation observed after 30 days (Figure S10). This is likely due to their ability to form hydrogen bonds between the lignin hydroxyl groups and the ether groups of M_1_.

By contrast, poor dispersibility of LGK, LGOH, and LGD-A samples was already observed within just 3 h after vortex mixing and after 24 h for LGO-A. Despite having a high number of OH groups, the poor dispersibility of Kraft lignin in the acrylic resin could be attributed to its high molecular weight (*M*_w_ = 6000 g·mol^–1^, as reported by the manufacturer) and high sulfur content (1.56%, determined by our elemental analysis). The reduction in –OH groups capable of interacting with the ether groups of M_1_, coupled with a higher tendency to agglomerate, may account for the rapid sedimentation observed for hydroxyalkylated and acrylated lignin.

Consequently, LGK and hydroxyalkylated lignin were excluded from further analysis, whereas acrylated lignin were retained considering the reported benefits for printed composites.^12,17,20–22^ Subsequent analyses focus on comparing the acrylated and nonacrylated forms of LGO and LGD lignin.

#### 3.2.2 Viscosity of Uncured Formulations

The viscosity of uncured formulations, identical to those used in 3D printing, was evaluated considering the influence of photoinitiators and fillers. Both the concentration of LGO and BAPO (Figure S11) and type of lignin at 2 wt % (Figure S12) were examined. All formulations exhibited shear-thinning behavior at low shear rates, stabilizing around 10 s^–1^ as non-Newtonian fluids, typical of acrylic formulations composed of low molecular weight monomers.^11^

Viscosity increased with LGO content (Table 1), due to restricted polymer chain mobility induced by lignin particles.^12^ At 2 wt % lignin, LGO2, LGD2, LGO-A2 and LGD-A2 increased viscosity relative to the neat acrylic resin by factors of 2.7, 3.4, 1.4 and 2.3, respectively. The higher viscosity of LGD2 is attributed to its larger particle size, whereas the lower viscosities of acrylated lignin formulations likely result from poorer compatibility with the acrylic matrix, as evidenced by sedimentation tests. Anyhow, all formulations showed viscosities substantially below those of commercial resins (0.2–4.5 Pa·s).^44^ Viscosities below 2 Pa·s are generally preferred to ensure adequate flow into the narrow gap between the cured layer and the resin tray,^45^ although excessively low viscosities can induce cracking due to shrinkage stress.^46^ Thus, small lignin additions may be advantageous for very low viscosity formulations such as the neat acrylic resin used in this study.

#### 3.2.3 UV-Absorption of Lignin

The UV–vis spectra of the lignin extracts were recorded either in THF solution (Figure S13), as well as in the neat acrylic resin as suspensions (Figure S14). The absorptivity values in solution at 405 nm were also calculated except in acrylated lignin due to solubility problems (Table 2). In general terms, LGK exhibited the lowest absorptivity, followed by LGO, while LGD showed the highest value, indicating that the DES extraction process may negatively affect the photopolymerization rate.^11^ Concerning acrylation, the absorption value at 405 nm of LGO-A remained unchanged relative to LGO, within experimental error. By contrast, the UV absorption at 405 nm of LGD-A significantly decreased compared with LGD (Figure S14). Previous studies have also reported significant reductions in lignin absorptivity after acetylation combined with reduction.^17^

Overall, the results provide an initial insight into the potential influence of lignin fillers on the photopolymerization rate.^11,24^ Based on the UV absorptivity values, the photoinitiator concentration was matched to that of lignin at a 1:1 ratio, except for LGD, where a 3:1 ratio was applied. Cure depth tests confirmed the validity of this approach.

#### 3.2.4 Cure Depth Tests

The curing properties of the formulations were compared by calculating *D*_p_ and *E*_c_ through Jacob’s working curves^33^ (Table 1, Figure S15). *D*_p_ decreased 2.8- and 8.7-fold for LGO1 and LGO2, and 8.7- and 15-fold for LGD1 and LGD2, respectively, consistent with lignin’s UV light absorption and free radical scavenging activity compared to the neat polymer. Additionally, *D*_p_ values for LGO composites declined exponentially with increasing lignin content up to 4 wt %. The higher UV absorptivity of LGD restricted its useable concentration to 2 wt %. To some extent, a lower *D*_p_ can be advantageous, as it produces a dye-like effect that confines polymerization to the intended layer, thereby improving print resolution and dimensional accuracy.^13^ For a fixed photoinitiator concentration, a reduction in *D*_p_ would typically correspond to an increase in the *E*_c_. Nonetheless, as the photoinitiator content was increased proportionally with lignin, free radical generation was enhanced and could offset or even reduce *E*_c_. This outcome demonstrates that additional BAPO effectively compensates for lignin’s UV absorption and improves overall reactivity.

In agreement with previous reports, acrylated lignins significantly improved curing behavior by increasing *D*_p_ and decreasing *E*_c_. The effect was more pronounced for LGD-A, owing to its markedly lower absorptivity at 405 nm compared to LGD: *D*_p_ increased by approximately 2.7-fold, whereas *E*_c_ decreased to half its original value. These improvements arose from both the reduced UV absorptivity and the presence of additional reactive acrylate groups.^12,20^ Notably, the enhanced photoreactivity of LGD-A enabled the use of only one-third of the initiator concentration required for its nonfunctionalized counterpart (2 wt % vs 6 wt % BAPO for R-LGD2 and R-LGD-A2, respectively).

### 3.3 Characterization of Printed Composites

#### 3.3.1 FTIR Analysis and Yield of Insoluble Fraction

The ATR-FTIR spectra of the neat acrylic resin and 2 wt % lignin composites were analyzed before and after DLP printing to confirm the final degree of double bond conversion (% DBC) in the as-printed samples^47^ and possible lignin-polymer interactions (Figure 1A). The ATR-FTIR spectra of the lignin composites closely overlap with that of the neat acrylic resin, suggesting that incorporation of lignin at low concentrations did not alter the chemical structure of the acrylate matrix. Subtle variations in the bands associated with the (meth)­acrylate C=C vibrations were evident, pointing to incomplete polymerization. Specifically, the bands at 1640 cm^–1^ (C=C stretching, ν_C=C_), 1407 cm^–1^ (in-plane deformation, scissoring, δ_=CH2_), and 813 cm^–1^ (out-of-plane deformation, δ_=CH2_) were identified. Nevertheless, as shown in Table S1, high degrees of double bond conversion were achieved (% DBC = 90 to 94.5%), with the highest value observed for the composite containing the greatest BAPO concentration (R-LGD2).

**Figure 1:**
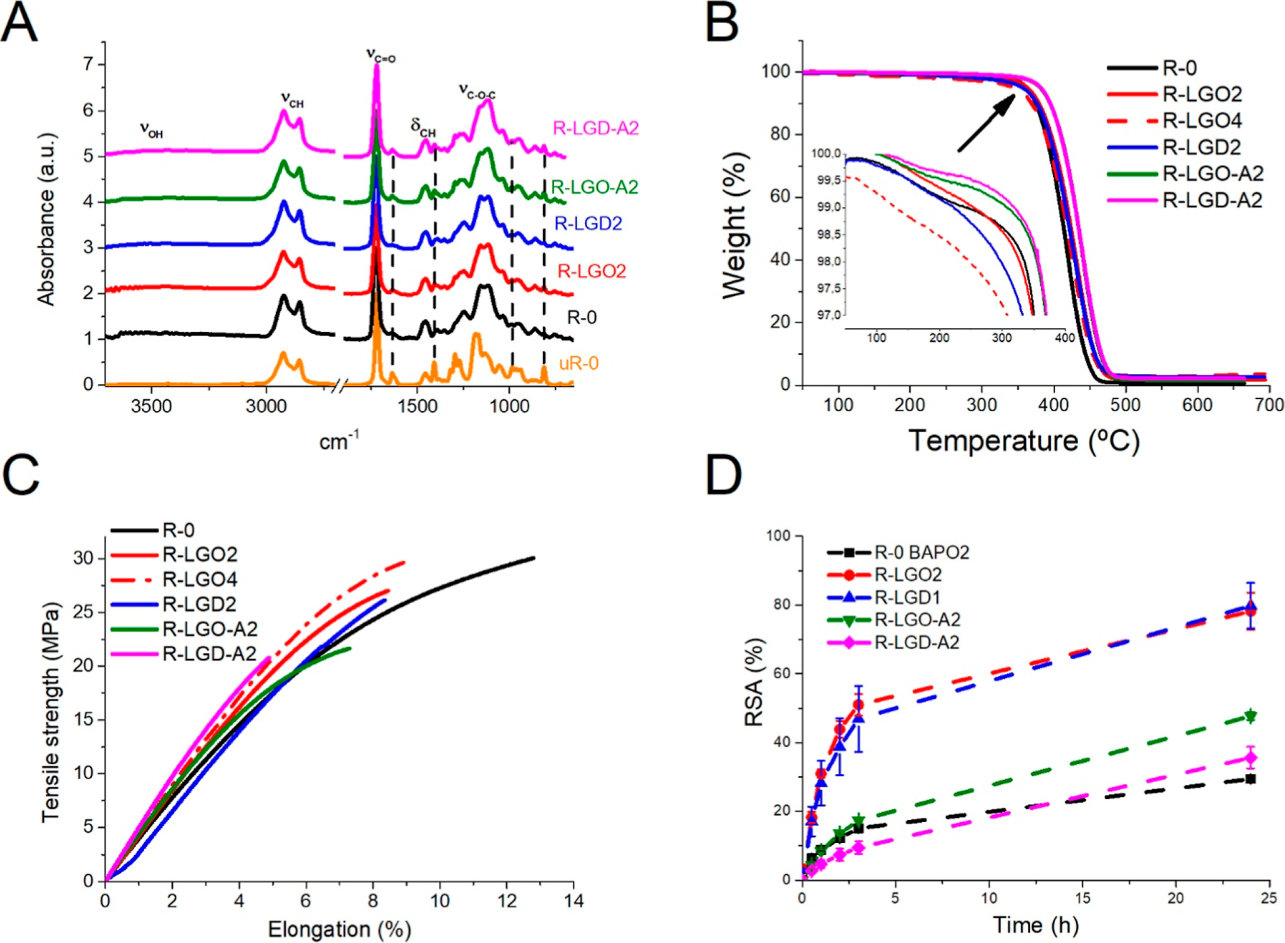
ATR-FTIR spectra (A), TGA curves (B), stress–strain behavior (C), and radical-scavenging activity (% RSA) (D) of the neat acrylic resin and lignin composites. The ATR-FTIR spectra were normalized to the carbonyl stretching band at 1728 cm^–1^. Dashed lines indicate characteristic (meth)­acrylate C=C bands, which decrease upon photopolymerization.

Consistent with the FTIR results, the formation of highly cross-linked structures was further supported by the yield of insoluble fraction (YIF %) following acetone extraction of the neat acrylic resin and 2 wt % lignin composites (Table S1). Samples R-0, R-LGO2, R-LGO-A2 and R-LGD-A2 exhibited YIF values of 99 ± 1%, while R-LGD2 showed a slightly lower value of 97%, likely due to the partial solubility of DES lignin in acetone. These findings contrast with previous reports that observed a marked decrease in gel content and cross-linking degree with increasing nonfunctionalized lignin content in the composites.^14^ Overall, the results confirmed that increasing the photoinitiator concentration and extending the light curing time, as predicted from cure depth tests, effectively counteract the adverse effects of lignin’s UV absorption.

#### 3.3.2 Dynamic Mechanical Analysis (DMA)

Figure 2 displays the log of the storage modulus (*E*′) and tangent delta (tan δ) curves of the neat acrylic resin and 2 wt % lignin composites obtained by DMA as function of temperature. Table S1 summarizes the average values of the storage moduli at selected temperatures, molecular weight of chain segments between cross-links (*M*_c_), cross-link density (υ_c_), and glass transition temperatures (*T*_g_) calculated from the curves plotted in Figure 2.

**Figure 2:**
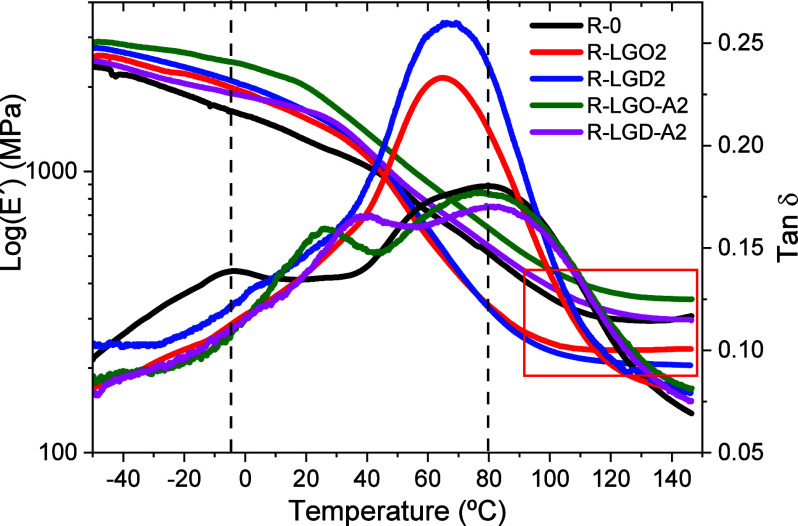
Effect of sample composition on the temperature dependence of the log storage modulus (*E*′, left *Y*-axis) and loss tangent (tan δ, right *Y*-axis) measured at 1 Hz.

The log of the storage modulus curves of both the neat acrylic resin and 2 wt % lignin composites exhibited a gradual decline over a broad temperature range without a well-defined plateau in the glassy region. This behavior is characteristic of highly cross-linked and heterogeneous polymer networks with broad relaxation distributions.^47^ The storage modulus decreases progressively from approximately −50 to 30 °C, and the decrease becomes more pronounced above 30–40 °C, depending on the formulation, marking the onset of an extended glass–rubber transition region. The modulus reaches a minimum near 120 °C (rubbery plateau region, indicated by the box in Figure 2). No postglass-transition increase in *E*′ was detected, indicating that no further cross-linking occurred at elevated temperatures.^48^

The tan δ vs temperature curve of the neat acrylic resin, exhibited two separate broad damping peaks: the glass transition at 81 °C and a β transition at 4 °C. β-relaxations are associated with the movement of side groups or small segments of the main polymer chain rather than the entire backbone.^49^ The glass transition was intermediate with respect to the individual components (116 and 58 °C for p-M_1_ and p-M_2_, respectively), which confirmed the formation of a random copolymer (Figure S16, Table S1). Similar two-glass transition profiles in tan δ curves are typically observed in highly cross-linked polymers, which exhibit broad glass transition regions owing to a range of molecular environments in which a variety of chain units are found.^50^

Consistent with the reduced cross-linking densities, the presence of native lignin decreased the glass transition temperatures (*T*_g_) of R-LGO2 and R-LGD2 by 15 and 12 °C relative to R-0, respectively. Moreover, the tan δ peak heights of R-LGO2 and R-LGD2 increased, reflecting higher viscous behavior, greater energy dissipation, and weaker filler–matrix interfacial adhesion. Similar decreases in rubbery modulus and *T*_g_ at low lignin loadings have been previously reported and were attributed to increased free volume and enhanced segmental mobility associated with reduced cross-link density^51,52^ as well as to the plasticizing effect of lignin soluble fractions.^53^

In addition to changes in global cross-link density, the heterogeneous morphology of the composites, evidenced by the presence of lignin aggregates in SEM images, is expected to generate local variations in network structure at the particle–matrix interface. In systems containing native lignin, these poorly compatible and nonreactive domains likely disrupt the local *M*_1_/*M*_2_ copolymerization stoichiometry, producing interfacial regions with reduced effective network density and enhanced chain mobility.^54^

By contrast, although acrylated lignin also forms large aggregates, the *T*_g_ values and tan δ peak heights of R-LGO A2 and R- LGD A2 remained comparable to those of the neat acrylic copolymer. The cross-link density of R- LGO A2 showed a slight increase, while no significant variation was observed for R- LGD A2. Nevertheless, the pronounced increase in β transition temperatures (by 31 and 43 °C for R- LGO A2 and R- LGD A2, respectively) indicates restricted segmental mobility, consistent with a more elastic response. This behavior suggests that the presence of acrylate functionalities enables partial chemical integration of lignin at the aggregate–matrix interface, mitigating the formation of highly compliant interfacial regions despite the morphological heterogeneity.

Taken together, the DMA response of these materials reflects the combined effect of global cross-link density and localized network heterogeneities, with the final impact on *T*_g_ being strongly dependent on lignin reactivity rather than aggregate size alone.

#### 3.3.3 Thermogravimetric Analysis (TGA)

The thermal stability was evaluated by thermogravimetric analysis (Table S2). The lignin extracts exhibited a three-step weight loss behavior, described in the supplementary file (Figure S17). On the other hand, the neat acrylic resin showed one-step weight loss behavior and the degradation profiles of the lignin composites were nearly identical to that of the neat acrylic resin, with no detectable volatile evaporation, consistent with the high double-bond conversion determined by FTIR (Figure 1B).

Composites containing unmodified lignin showed no significant changes in the maximum degradation temperature. Only slight decreases in *T*_10_ and *T*_onset_ were observed in agreement with the lower thermal stability of lignin compared to the acrylic matrix. The early degradation of lignin may contribute to protecting the acrylate matrix, as all composites remained thermally stable up to 370 °C.^55^ By contrast, the inclusion of 2 wt % of acrylated lignin notably increased the thermal stability of the composites in terms of *T*_10_, *T*_onset_, *T*_max_, indicating higher cross-link density compared to composites containing 2 wt % unmodified lignin, in coherence with the DMA results.

#### 3.3.4 Mechanical Properties and Fracture Surface Morphology

The uniaxial tensile and Shore D hardness results for composites containing various lignin types and loadings, in comparison with the neat acrylic resin are presented in Figure 1C and Table S3. As expected for a random copolymer, the neat acrylic resin exhibited intermediate mechanical behavior relative to its individual monomeric components. It showed a high Young’s modulus and high tensile strength, reflecting strong resistance to deformation under tensile loading due to its 50 wt % dimethacrylate content and the resulting high cross-linking density.

The addition of 1 and 2 wt % of nonfunctionalized organosolv lignin did not significantly alter the Young’s modulus but decreased both tensile strength and elongation at break, resulting in a reduction in tensile toughness. A similar behavior was observed for the DES-derived lignin composites (R-LGD1 and R-LGD2). This mechanical equivalence suggests that the potentially adverse influence of the larger DES lignin particles was counterbalanced by their better compatibility with the resin, as supported by sedimentation tests^56^ and by the increase in the number of sonication cycles.

Interestingly, the decline in tensile properties was partially reversed at 3 and 4 wt % organosolv lignin loadings, such that the toughness of R-LGO4 reached approximately 35% of the R-0 value. These observations differ from commonly reported trends, in which increasing nonfunctionalized lignin content typically deteriorates mechanical properties through particle aggregation, reduced cross-link density, or interfacial debonding.^11,13,16,52^ In this work, adjusting the photoinitiator concentration proportionally to the lignin content likely mitigated such effects.

Moreover, the hardness of all R-LGO and R-LGD composites increased, consistent with the role of lignin particles as rigid fillers,^15,16^ despite the slight reduction in cross-linking density observed by DMA.

In contrast, acrylated lignin did not yield the anticipated reinforcing effect.^12,17,18,20,22^ Incorporation of 2 wt % acrylated lignin notably decreased tensile strength (≈39%) and elongation at break (51% and 62%, for R-LGO-A2 and R-LGD-A2, respectively), along with minor increases in Young’s modulus, and hardness values statistically comparable to the neat resin. This outcome was unexpected, as acrylation was intended to improve compatibility with the hydrophobic component of the resin and, consequently, enhance the mechanical performance. However, sedimentation tests revealed phase separation, suggesting limited interfacial adhesion despite chemical modification, potentially promoting aggregate formation. Moreover, acrylation increased the cross-linking density compared to 2 wt % nonfunctionalized lignin composites, as confirmed by DMA analysis, ultimately contributing to reduced tensile toughness.

SEM micrographs of the fractured cross sections of 2 wt % lignin composites after tensile testing provide valuable insight into their fracture behavior compared to the neat resin (Figures 3 and S18). No evidence of interlayer voids or delamination was detected in any sample, confirming that all formulations achieved dense and well-cured network structures.^57^ The fractured surface of the neat resin exhibits two distinct regions (Figures 3A, and S18A,B): a rough area characterized by irregular protrusions and wedge-shaped river-like features aligned with the fracture direction and a smooth, flat zone displaying fine, radially oriented lines. This image is consistent with a mixed brittle–ductile fracture mode.^58^

**Figure 3:**
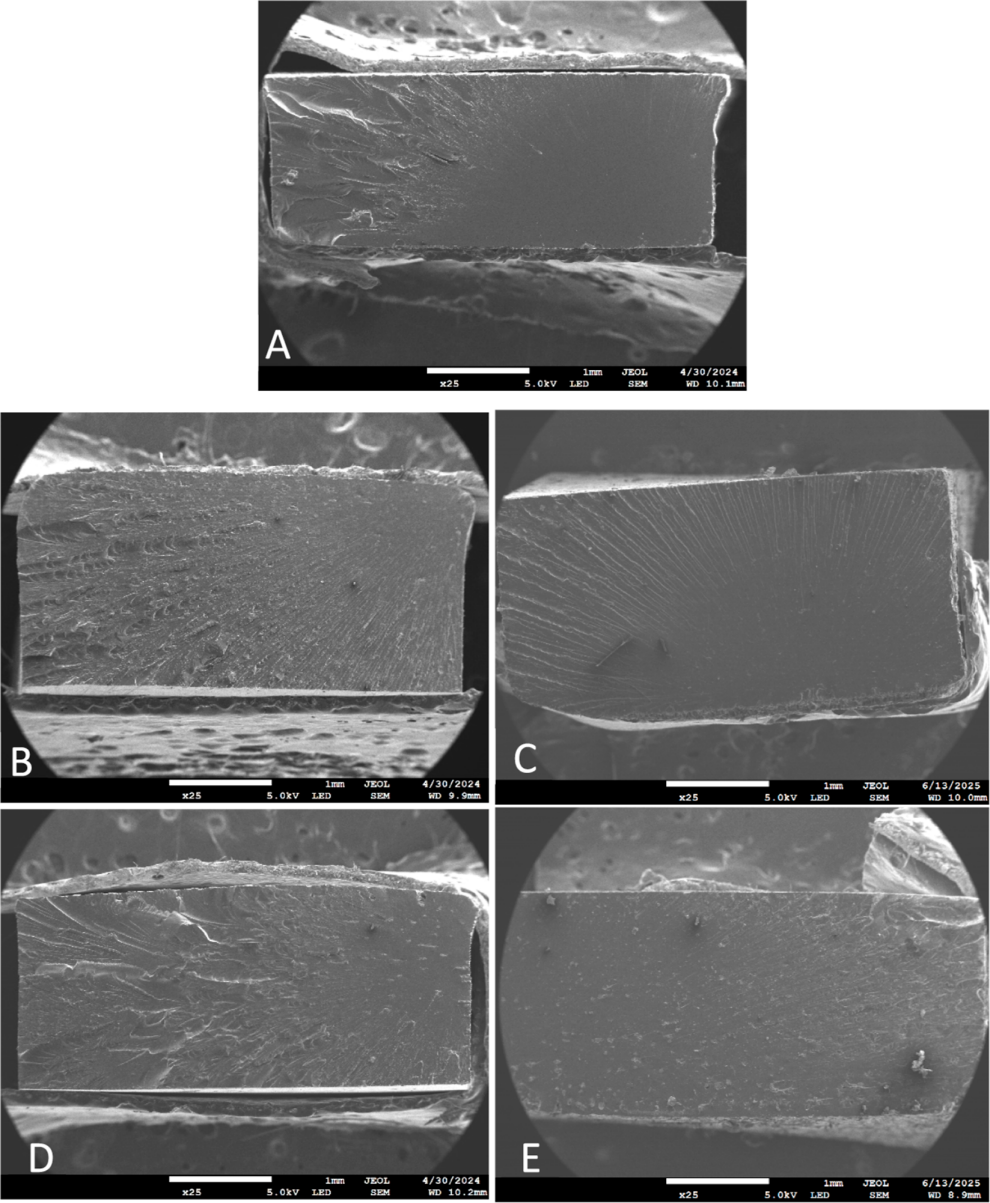
SEM images of tensile fracture cross sections at 25× magnification: (A) R-0, (B) R-LGO2, (C) R-LGO-A2, (D) R-LGD2, and (E) R-LGD-A2.

The incorporation of native lignin increased the roughness of the fracture cross sections of R-LGO2 and R-LGD2 (Figure 3B,D), suggesting a shift toward a less brittle, more energy-dissipative fracture mechanism. At higher magnification, sharp edges and sawtooth-like patterns become evident (Figure S18C,E). Micron-sized lignin agglomerates appear uniformly distributed across the fracture surface and may act as stress concentrators or crack initiation sites. Further, composites incorporating acrylated lignin (R-LGO-A2 and R-LGD-A2) displayed smoother fracture areas with more numerous and larger aggregates (Figures 3C,E; and S18D,F). These features are characteristic of more brittle fracture. The presence of large, poorly dispersed lignin domains suggests incomplete solubility and suboptimal interfacial bonding, which hinder efficient stress transfer across the matrix.

#### 3.3.5 Water Swelling and Surface Properties

The water-related properties of the printed lignin composites were evaluated because lignin can influence swelling, hydrolytic stability, and wettability. Water equilibrium swelling was achieved after 30 days of immersion. With the exception of R-LGO-A2, the 2 wt % lignin composites (1.90–2.11%) exhibited slightly but significantly higher swelling than R-0 (1.71% ± 0.04). Increasing lignin loading from 2 to 4 wt % in the R-LGO series did not affect swelling behavior. After drying, all samples showed minimal mass loss (≥−0.23%), confirming excellent dimensional stability and hydrolytic resistance.

Lignin also modified surface properties, as shown in Table 3. Surface roughness decreased by 82–84% in composites containing unmodified lignin, with no significant differences between R-LGO2 and R-LGD2 (*p* > 0.05). In contrast, roughness reductions were limited to 44% and 22% in the acrylated lignin composites R-LGO-A2 and R-LGD-A2, respectively. These changes explain the glossy appearance of LGO2 and LGD2, compared with the matte finish of R-0, LGO-A2, and LGD-A2.

**Table 3:** Mean Water Contact Angles, *S*_a_ (Arithmetic Mean Roughness), *S*_q_ (Root Mean Square Roughness) of the Neat Acrylic Resin (R-0) and Composites Containing 2 wt % Lignin, and Circularity of Porous Square Plates with Circular Holes

sample	water contact angle (°)	*S*_a_ (μm)	*S*_q_ (μm)	circularity
R-0	81 ± 2	1.32 ± 0.05	1.93 ± 0.09	0.88 ± 0.08
R-LGO2	75 ± 1	0.21 ± 0.03	0.28 ± 0.04	0.93 ± 0.03
R-LGD2	70 ± 4	0.24 ± 0.06	0.31 ± 0.08	0.93 ± 0.02
R-LGO-A2	77 ± 1	0.74 ± 0.06	1.10 ± 0.14	0.91 ± 0.06
R-LGD-A2	75 ± 5	1.03 ± 0.08	1.71 ± 0.47	-

The influence of lignin on wettability depends on both lignin type and polymer matrix. In some systems, lignin increases wettability,^11^ while in others, it reduces it.^59^ Both nonacrylated and acrylated lignin decreased water contact angles (CA), with a stronger effect for the former. These trends are not attributable to surface topography, as S_a_ values were too low to markedly influence wettability.^60^ Instead, the reductions reflect the intrinsic hydrophilicity of lignin, particularly native lignin, enriched in polar carboxyl and phenolic groups. Acrylated lignin composites displayed slightly higher CAs than their nonacrylated counterparts, reflecting the loss of hydrophilic functionalities during acrylation.

In a nutshell, surface properties strongly depend on lignin type. Small additions of nonfunctionalized lignin more effectively enhance surface finish, yielding smoother, glossier, and more hydrophilic surfaces, than acrylated lignin. Improved wettability may also facilitate layer adhesion during printing and benefit applications requiring fluid interaction, such as antistatic coatings, electrochemical sensors, and microfluidic devices.^61^

#### 3.3.6 Printability

High reactivity and rapid curing times of acrylic resins come along with certain drawbacks, such as reduced dimensional accuracy, shrinkage and curling.^45^ Low lignin contents can improve both surface roughness and print resolution, despite its UV light absorption and scattering effects.^13,17,18^ The print quality of selected 2 wt % lignin formulations was analyzed by using two representative 3D models with circular or square holes (Figures 4 and S19).

**Figure 4:**
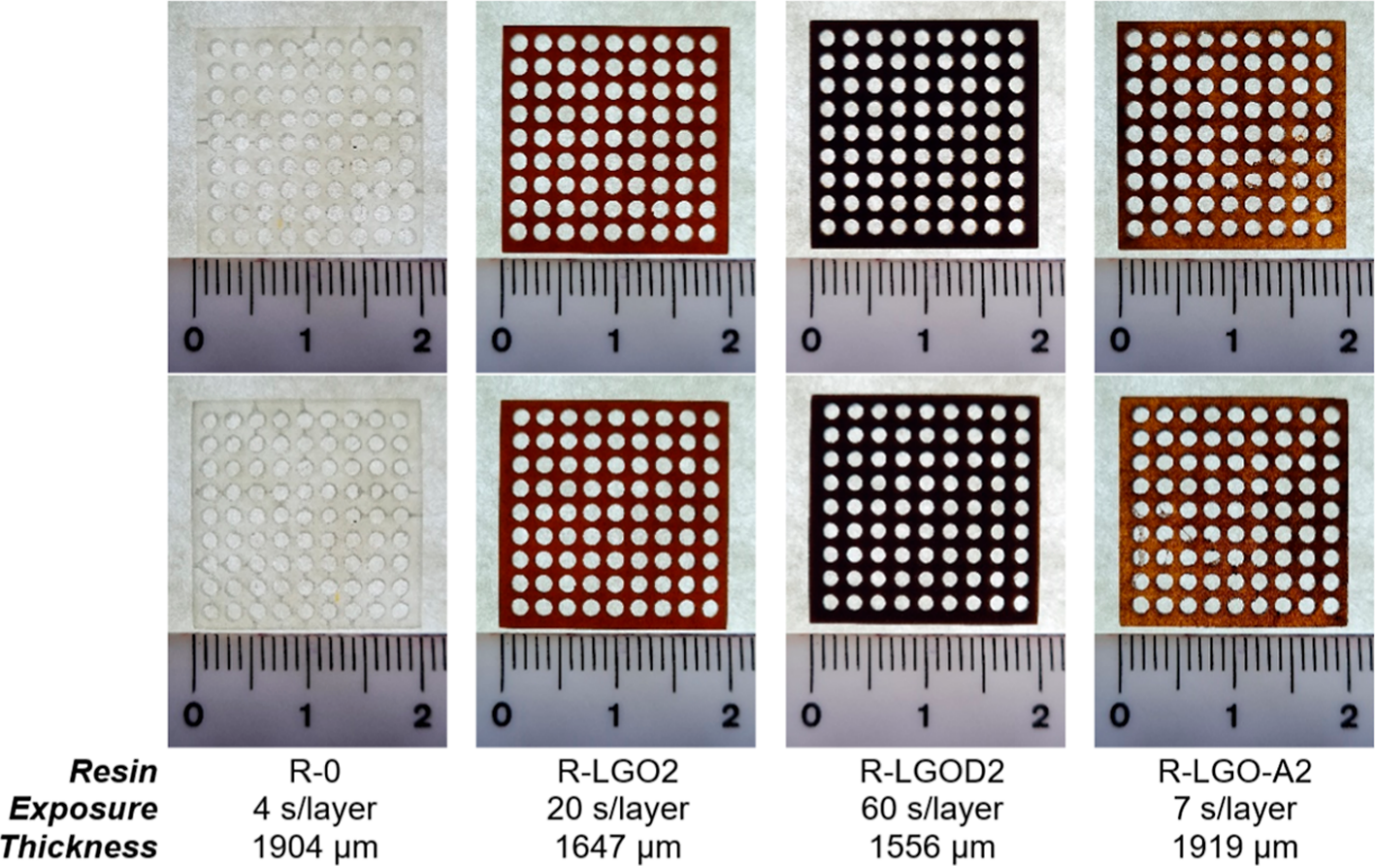
Images of porous-structured square plates with circular holes, printed with the neat acrylic resin (R-0) and selected 2 wt % lignin composites. Images were captured with a cell-phone camera at 3× magnification. The theoretical dimensions of the plate model were set to 20.02 × 20.02 × 2.00 mm, with circular hole diameters of 1.40 mm. Top row—front side; bottom row—back side. Ruler units are expressed in centimeters.

In both models, the neat resin visually showed pronounced inaccuracies of the uniformity of the holes, including multiple surface defects on both sides such as cone-like deformations for cylindrical holes, and many closed square holes. Conversely, the samples containing lignin fillers showed no visible defects on either side, and the hole boundaries were well-defined. An exception was noted in the square-porous plates printed with R-LGD2, which displayed partially closed and poorly defined square holes attributed to the longer polymerization time required and excessive over curing of each layer.^62^ The circularity values (Table 3) calculated for the circular holes according to eq 1 showed no significant differences among the lignin composites, all of them exceeded 0.90 when close to 1 indicates a perfect circle.

Regarding the dimensions along the *x*, *y*, and *z* axes of the 3D-printed porous plates, the *x* and *y* dimensions of all formulations matched the nominal value (20.0 ± 0.1 mm) while differences were found for the thickness. Neat resin and R-LGO-A2 exhibited thickness values closest to the theoretical 2.00 mm, whereas for R-LGO2 and R-LGD2 it decreased by 18% and 22%, respectively (Figure 4). These deviations in the *z*-axis are attributed to excessive overcuring in these two samples that is particularly evident in porous structures.^63^

Consistent with previous research,^16^ incorporating 2 wt % lignin fillers significantly improved detail in the printed structures. Nevertheless, certain printing parameters must be carefully adjusted and fine-tuned dependingon the filler’s UV absorptivity and particle size, in accordance with the desired geometry and intended application of the printed object. To achieve optimal results, cure-depths studies are essential, and process parameters, particularly those related to overcuring, should be customized to maximize detail refinement.

#### 3.3.7 Antioxidant Activity

The antioxidant properties of lignin-based 3D-printed composites were assessed using the DPPH assay.^64^ To decouple the effect of lignin type from other variables, such as photoinitiator concentration or residual (meth)­acrylate groups, neat acrylic resins containing varying photoinitiator concentrations were also examined (Figure S20). All samples showed an exponential increase in antioxidant activity, reaching a plateau at 24 h. Given the substantial contribution of BAPO at high concentrations, R-0-BAPO-4 (84.1 ± 1.6% at 4 wt %), R-LGO4 (4 wt % BAPO) and R-LGD2 (6 wt % BAPO) were excluded from further analysis.

As seen in Figure 1D, composites containing 1–2 wt % nonacrylated organosolv lignin exhibited markedly higher antioxidant activity than the neat acrylic resin, showing ∼78% greater RSA after 24 h. No statistically significant differences were observed between R-LGO1 and R-LGD1, despite differences in photoinitiator contents and lignin structures (e.g., phenolic and aliphatic hydroxyl groups, methoxyl units, particle size).^42,64^ As expected, lignin acrylation substantially reduced antioxidant activity. After 24 h, the RSA of R-LGO-A2 decreased from 76 ± 4% (R-LGO2) to 46 ± 1%, while the RSA of R-LGD-A2 was nearly eliminated, matching that of neat acrylic resins with equivalent BAPO levels. This decline reflects the extensive depletion of free phenolic hydroxyl groups upon acrylation (Table 2).

The RSA values for R-LGO1, R-LGO2, and R-LGD1 after 3 h were comparable to those reported by Ruiz Deance et al. for methacrylated Kraft lignin–PEGDA 575 composites, although their measurements exhibited higher uncertainty, due to the use of shredded SLA-printed parts.^22^ At any rate, their results were noteworthy despite >99.5% conversion of Kraft lignin aromatic hydroxyl groups to methacrylate esters. To the authors’ knowledge, aside from that study, no other researchers have explored the antioxidant activity of lignin composites produced by vat-photopolymerization. Nonetheless, the values observed here align with those of other lignin-containing composites, including PLA-based systems fabricated by both additive and subtractive methods.^65,66^

In short, the nonacrylated-lignin composites produced in this work exhibited strong antioxidant activity at low lignin loadings, regardless of the extraction method. Their radical scavenging activity approaches that of commercial antioxidants,^64^ highlighting their potential for applications in healthcare, electronics and related fields.

## 4 Conclusions

This study systematically investigated the incorporation of organosolv (LGO) and deep eutectic solvent-derived (LGD) lignin, in both native and acrylated forms, into a newly formulated partially biobased acrylic resin for DLP. The suitability of the two approaches, nonchemical modification versus chemical functionalization, was discussed from various perspectives.

Comprehensive characterization of lignin physicochemical properties, dispersion stability, and UV absorptivity revealed their critical influence on resin processability. Particle size distribution and chemical compatibility with the acrylic copolymer were key factors in preventing phase separation and sedimentation during printing. Ultrasonication and vortex mixing cycles were increased prior to printing to address the issue of large particle size. A high conversion rate was maintained in the lignin composites by adjusting the overcure ratio and the photoinitiator concentration considering the UV absorption.

The type of lignin selected would ultimately depend on the physicochemical compatibility with a particular photopolymerizable formulation and desired end-use properties. The addition of nonfunctionalized lignin at low to moderate loadings (LGO, LGD) provided excellent dispersion stability, enhanced print resolution, improved surface finish, tunable wettability and showed effective antioxidant capacity, but acted as a plasticizer and reduced *T*_g_ and cross-linking density. Moreover, they slightly enhanced Young Modulus and hardness of the acrylic matrix with losses in tensile toughness at 1 and 2 wt % than can be partially recovered at LGO moderate loadings (3 and 4 wt %). Chemically functionalized acrylated lignin (LGO-A, LGD-A) improved curing behavior, increased cross-link density, thermal stability, and maintained *T*_g_ by integrating into the network, but led to poorer tensile performance and more brittle fracture due to limited interfacial compatibility and aggregation. Moreover, antioxidant function was lost due to the depletion of phenolic hydroxyl groups.

These results underscore the complex interplay between lignin structure, reactivity, and morphology in defining composite behavior and highlight key parameters for optimizing lignin-formulations for additive manufacturing applications. Collectively, the results highlight a trade-off between structural integration and multifunctionality: while acrylation improved curing and network density, it compromised dispersion stability, toughness, and antioxidant activity, and increased material cost. In contrast, direct incorporation of unmodified lignin, particularly LGO, at 2–4 wt % offers a cost-effective and scalable route to multifunctional, high-resolution, and antioxidant-active resins providing a practical pathway for sustainable DLP 3D printing.

## Supplementary Material



## Notes

Supporting Information file (PDF) includes biorenewable carbon content (BRC, %) calculations for all acrylic formulations; description and ^31^P NMR spectra of acetosolv and DES lignin before and after hydroxyethylation and acrylation; description and ^13^C NMR spectra of acetosolv and DES native lignin and after acrylation and degree of substitution acrylation; figures showing the particle size distribution data for all lignin powders; descriptions and FTIR spectra of native and chemically modified lignin; an image demonstrating the stability of 2 wt % lignin dispersions in the photocurable resin; viscosity plots showing acetosolv lignin viscosity as a function of shear rate and lignin content and viscosity as a function of shear rate and lignin type at constant lignin content; UV–vis absorption spectra of lignin solutions in THF and lignin dispersions in the photocurable resin; DMA plots of logarithmic storage modulus (E′) and loss tangent (tan δ) versus temperature at 1 Hz for poly­(PEG200DMA), poly­(C10DA), and neat acrylic resin, together with a table of DMA parameters, double-bond conversion determined by FTIR, and yields after acetone extraction for the neat polymers and 2 wt % lignin composites; thermogravimetric analysis (TGA) of lignin extracts, including degradation profiles and a table of TGA parameters for lignin extracts, pure acrylic resin, and lignin composites; a table of tensile test parameters and Shore D hardness values for the neat copolymer and lignin composites; scanning electron microscopy (SEM) images of fractured cross sections of pure acrylic resin and lignin-based composites at 500× magnification; photographs of porous square plates produced from the neat acrylic resin and selected lignin composites; and plots of radical scavenging activity (RSA, %) as a function of photoinitiator content and lignin loading (PDF)

## ⊥

“Dunarea de Jos” University of Galati, Galati 800008, Romania
