# Supplementary material for: Tailoring Renewable Photopolymers with Lignin: Printability, Surface and Antioxidant Properties
Source: ACS Appl Polym Mater. 2026 Apr 24;8(9):6645–58. doi: 10.1021/acsapm.6c00559 (PMC13162294; doi:10.1021/acsapm.6c00559)
Supplement: Supplementary file 1 [file ap6c00559_si_001.pdf]

Supporting Information

Tailoring Renewable Photopolymers with Lignin: printability, surface and antioxidant properties

Marius Bodor[1,†], Aurora Lasagabáster-Latorre[2], Pablo Ligero[3], Sandra María García-Garabal[3], María Luisa Sánchez Simón[4], Sonia Dopico-García[1], María-José Abad[1,]*

[1] Campus Industrial de Ferrol, CITENI-Grupo de Polímeros, Universidade da Coruña, Ferrol 15403, A Coruña, Spain

[2]Dpto Química Orgánica I, Facultad de Óptica y Optometría, Universidad Complutense de Madrid, Madrid 28037, Spain

[3] Facultade de Ciencias, BBP Group, Universidade da Coruña, A Coruña 15008, Spain

[4] Escuela Politécnica de Ingeniería, Campus Industrial de Ferrol, Universidade da Coruña, Ferrol 15403, A Coruña, Spain

[†] Current address: "Dunarea de Jos" University of Galati, Galati 800008, Romania

*Corresponding author.

María-José Abad: e-mail address: maria.jose.abad@udc.es

KEYWORDS. antioxidant activity, biobased polymers, lignin, photopolymers, 3D printing

1. BRC% content of acrylic formulations

The biorenewable carbon content (BRC%) of the composite formulations was calculated according to Equation 1:[1]

$$\text{BRC } \% = \frac{Biosourced\ Carbon}{Biosourced\ Carbon + Fossil} x100 \qquad (1)$$

According to technical data provided by Sartomer (Arkema, France), polyethylene glycol dimethacrylate (M_1= PEG200DMA) has a biorenewable carbon content of 50%, while 1,10-decanediol diacrylate (M_2= C10DA) has a biorenewable carbon content of 60%. The photoinitiator BAPO was considered to contain 0% biorenewable carbon. Lignin was assumed to contribute 100% biorenewable carbon; however, in the case of acrylated lignin, the actual biobased carbon content is expected to be slightly lower due to the incorporation of petroleum-derived acrylate moieties. The composition and calculated BRC% values of the prepared formulations are summarized in Table 1 of the main manuscript.

2. RMN of chemically modified lignins

Lignins obtained from the acetosolv and DES processes were first analyzed by ^{31}P NMR spectroscopy to quantify their aliphatic and aromatic hydroxyl group contents. Following hydroxyethylation, the ^{31}P NMR spectra revealed a pronounced increase in aliphatic hydroxyl groups (δ = 148–146 ppm), accompanied by a corresponding decrease in aromatic hydroxyl groups (δ = 144–137 ppm). This effect was more pronounced for DES lignin than for acetosolv lignin (Figure S1). The observed shift in hydroxyl group distribution indicates successful hydroxyethylation and suggests enhanced accessibility of aliphatic hydroxyl groups for subsequent grafting reactions.

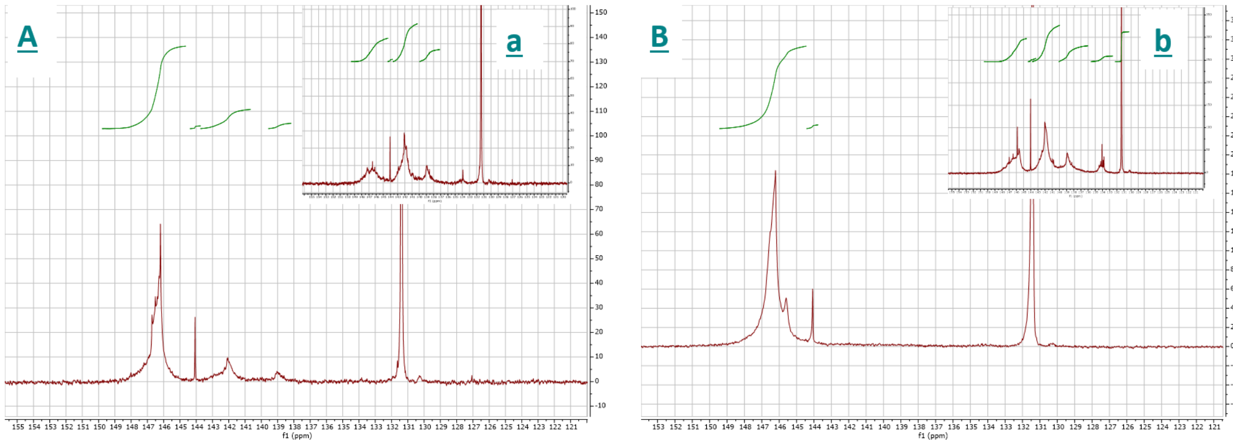

Figure S1. ^{31}P NMR of acetosolv (LGO) (left) and DES (LGD) (right) lignin before (a, b) and after (A, B) hydroxyethylation.

The hydroxyethylated acetosolv (LGOH) and DES (LGDOH) lignins were subsequently reacted with acrylic acid, and the extent of acrylation was first assessed by ^{31}P NMR spectroscopy. After acrylation, the ^{31}P NMR spectra (Figure S2) showed a strong reduction in aliphatic hydroxyl signals, corresponding to conversions of 83% for acetosolv lignin and 84% for DES lignin. In parallel, an increase in signals assigned to carboxylic acid groups (δ = 136–132 ppm) was detected. These signals are attributed to radical-mediated side reactions involving acrylic acid and vinyl groups of the modified lignin, as previously reported by Hua *et al.*[2] No resonances corresponding to unreacted acrylic acid were observed, indicating effective purification of the products.

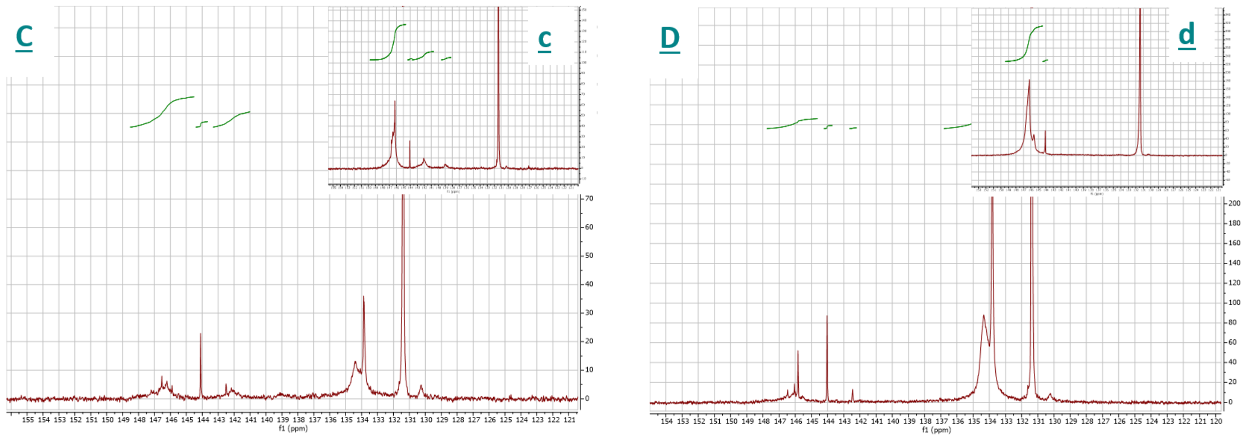

Figure S2. ^{31}P NMR of acetosolv (LGO) (left) and DES (LGD) (right) lignin before (c, d) and after (C, D) acrylation.

The successful incorporation of vinyl functionalities was further confirmed by ^{13}C NMR spectroscopy (Figures S3 and S4). Comparison of the full ^{13}C NMR spectra before and after acrylation revealed a decrease in signals associated with aliphatic carbon atoms bearing hydroxyl groups (δ = 59–61 ppm), together with the appearance of a new signal at δ = 63.6 ppm, assigned to ester-linked methylene carbons. In addition, the acrylated samples exhibited characteristic signals corresponding to the two carbon atoms of the vinyl group (δ = 127–132 ppm), as well as the ester carbonyl carbon (δ ≈ 165 ppm). These features were absent in the hydroxyethylated lignins, providing clear evidence of vinyl grafting.

The degree of substitution (DS) is defined as the ratio of esterified to total aliphatic hydroxyl carbons[2] and calculated according to Equation 2:

$$DS = \frac{A_{63.6}}{A_{59-61} + A_{63.6}} \qquad (2)$$

Where $A_{63.6}$ is the integrated area of carbon resonances of ester-linked methylene carbons after acrylation and A_{59-61} is the integrated area of carbon resonances corresponding to unreacted aliphatic hydroxyl groups.[2]

Finally, the ^{13}C NMR spectra of the acrylated lignins also displayed signals in the δ = 172–175 ppm region, attributed to carbonyl carbons from side products formed during reactions involving acrylic acid and lignin-derived vinyl groups. The relative intensity of these signals was higher for the DES-derived lignin (LGD-A) than for the acetosolv lignin (LGO-A), which is consistent with the stronger carboxylic acid signals observed in the corresponding ^{31}P NMR spectra. Overall, the combined ^{31}P and ^{13}C NMR analyses confirm the efficient acrylation of both lignins, with a higher extent of side reactions in the DES-derived material.

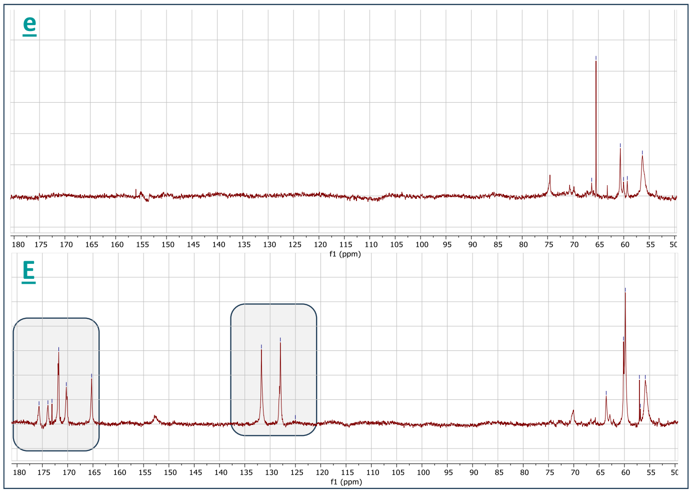

Figure S3. ^{13}C NMR of hydroxyethylated acetosolv lignin (LGOH) before (e) and after (E) reaction with acrylic acid

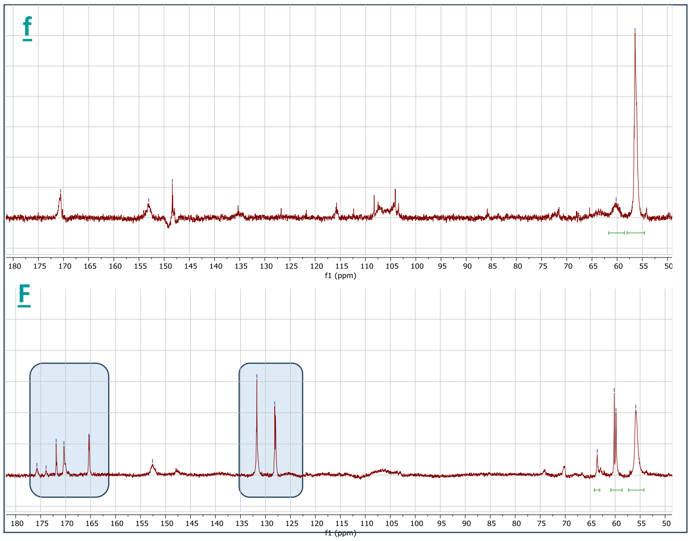

Figure S4. ^{13}C NMR of hydroxyethylated DES lignin (LGDOH) before (f) and after (F) reaction with acrylic acid

3. Particle size distribution

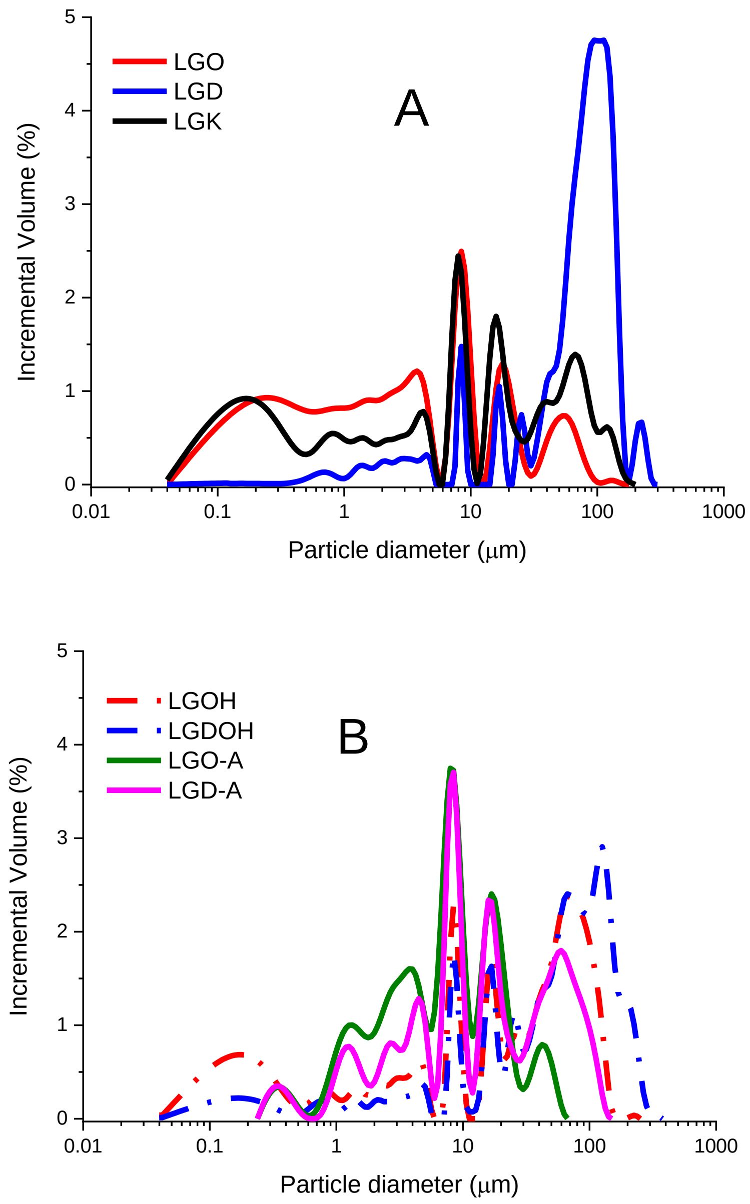

Figure S5. Distributive volume percent as a function particle diameter for lignin powders: (A) LGK, LGO, LGD, and (B) LGOH, LGDOH, LGO-A and LGD-A.

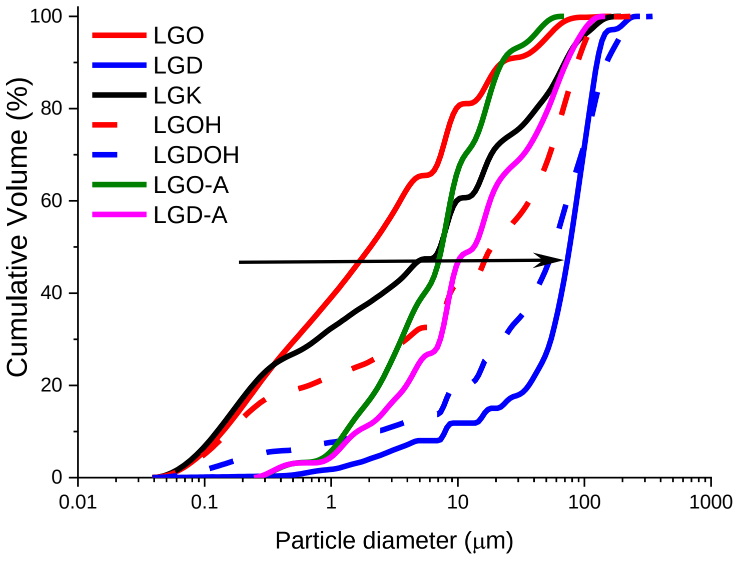

Figure S6. Cumulative volume percent as a function of particle diameter for all lignin powders.

4. FTIR-ATR spectra of native and modified lignin

The main differences between LGK and the two types of lignin extracted from Betula alba bark lie in the carbonyl stretching region and the fingerprint region (Figure S7). The bands assigned to the stretching vibrations of C=O groups unconjugated to aromatic rings, centered at 1732 and 1715 cm^{-1} in the LGO and LGD spectra, respectively, are stronger than the corresponding bands in the spectrum of LGK. A similar observation applies to the band at 1179 cm^{-1}, assigned to the C-O-C stretching vibrations of ester groups. Acid hydrolysis is promoted during LGO and LGD fractionation, generating phenolic hydroxyl and carbonyl/carboxyl groups.[3]

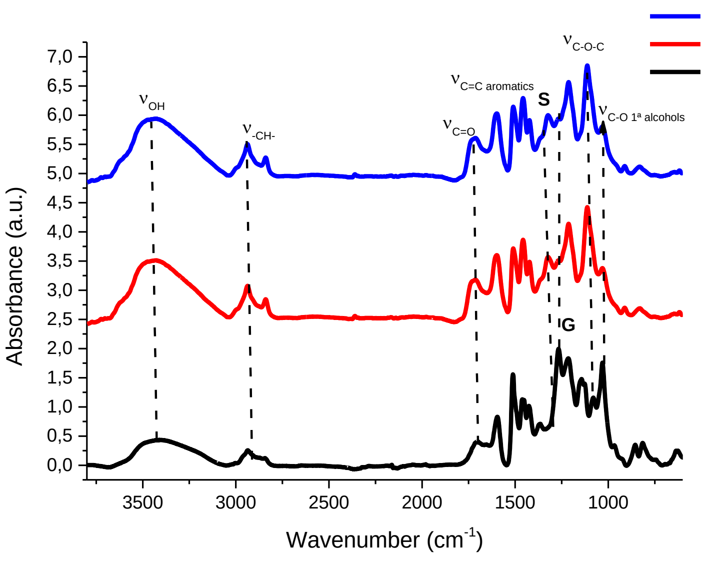

Figure S7. FTIR spectra of LGO, LGD and LGK. The spectra were normalized to the band of maximum intensity.

The effect of hydroxyalkylation can be observed in Figure S8. After hydroxyalkylation, a modest increase in the absorption band area centered at 3400 cm^{-1} (ν_{OH}) was observed, along with a notable increase in the intensities of the bands located at 2940 cm^{-1} (ν_{CHasym}), 2844 cm^{-1} (ν_{CHsym}), and 1030 cm^{-1} ($\nu_{C\text{-}O\ 1^{a}\ alcohol}$).

Figure S9 displays the FTIR spectra of LGO and LGD before and after acrylation. The spectra of the two acrylated lignin types (LGO-A and LGD-A) exhibit a significant reduction in the band corresponding to OH groups at 3400 cm^{-1} (ν_{OH}), together with a marked increase in the bands assigned to acrylate ester groups at 1770 cm^{-1} ($\nu_{C=O}$), 1184 cm^{-1} ($\nu_{C\text{-}O\text{-}C}$) and the in-plane bending vibrations of the =CH2 groups (808 cm^{-1}). As reported by previous authors, these spectral changes confirm the successful acrylation reaction at the OH groups.[2,4]

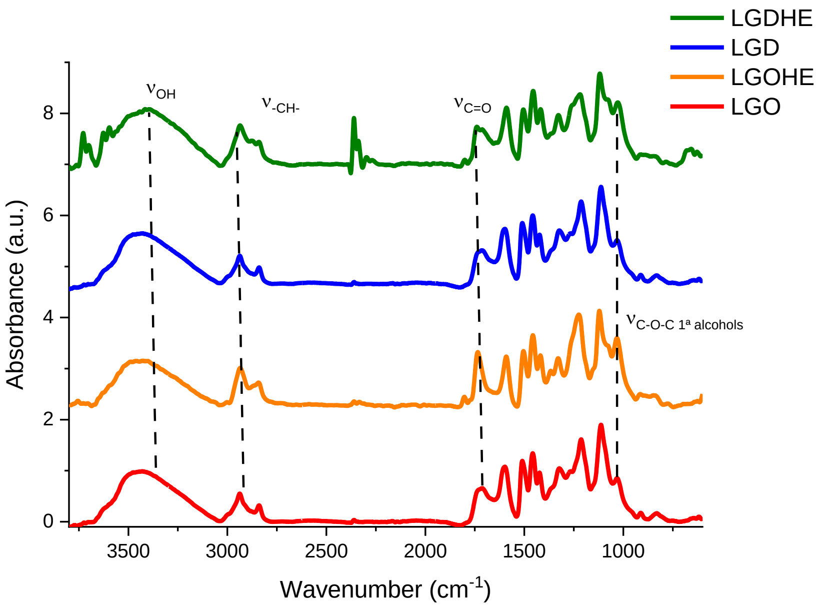

Figure S8. Effect of the hydroxialkylation reaction on the FTIR spectra of LGO and LGD. The spectra were normalized to the band of maximum intensity.

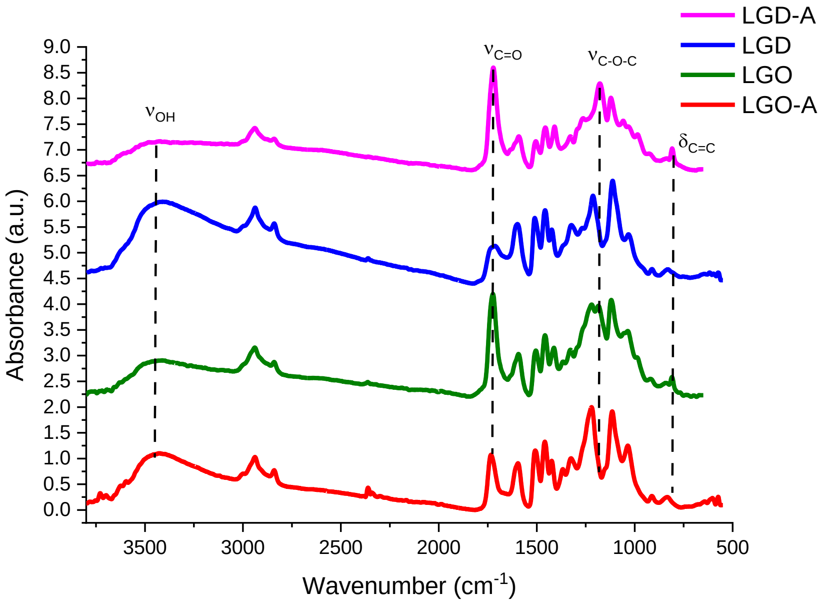

Figure S9. FTIR spectra of LGO and LGD lignin before and after acrylate modification. The spectra were normalized to the band of maximum intensity.

5. Dispersion stability of lignin in acrylic resin

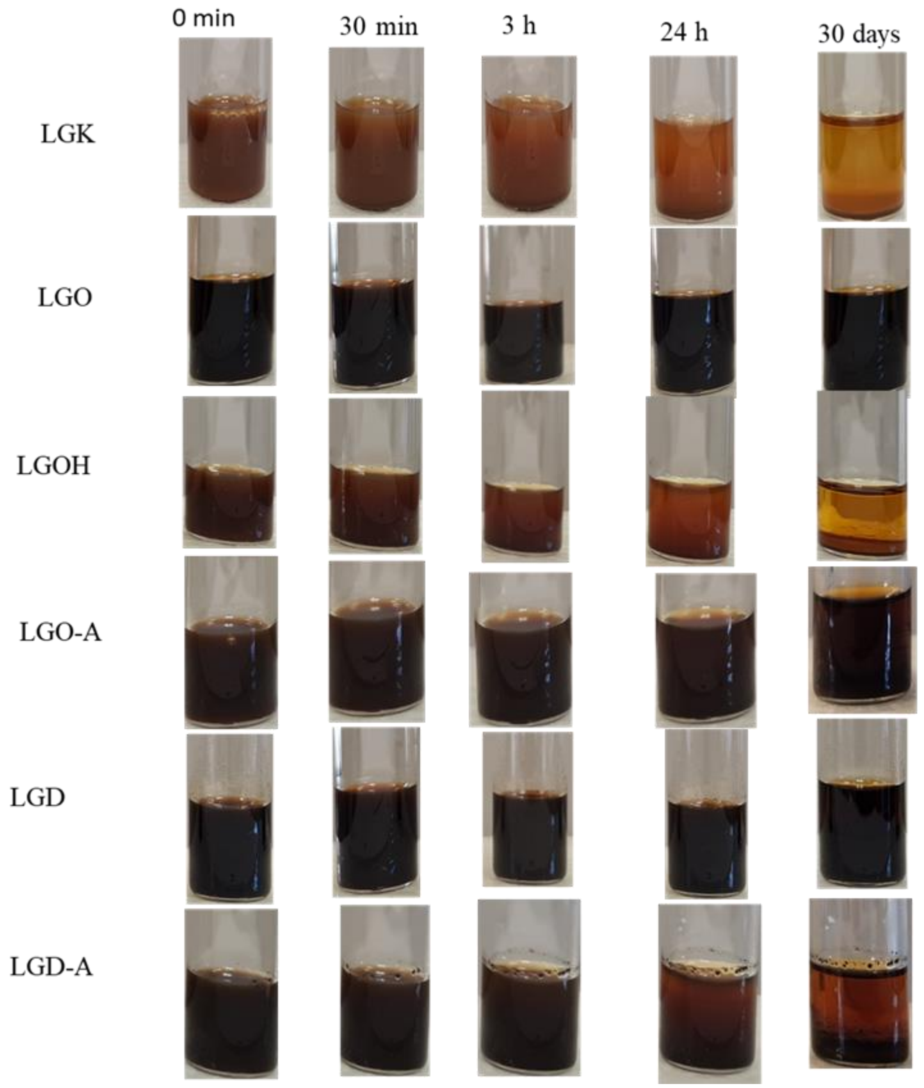

Figure S10. Evolution of 2 wt.% lignin dispersions in acrylic resin.

6. Viscosity of uncured formulations

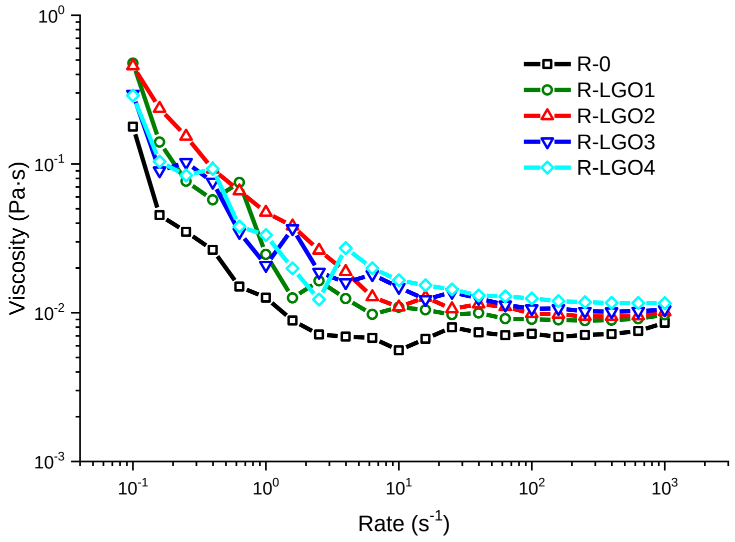

Figure S11. Viscosity as a function of shear rate, LGO and BAPO content at room temperature.

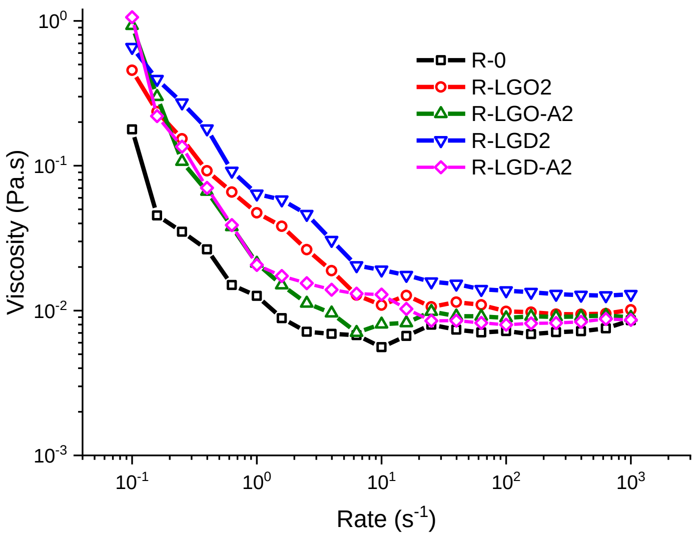

Figure S12. Viscosity as a function of shear rate and lignin type at room temperature, at a constant lignin content of 2 wt.%.

7. UV-vis spectra of selected lignin types

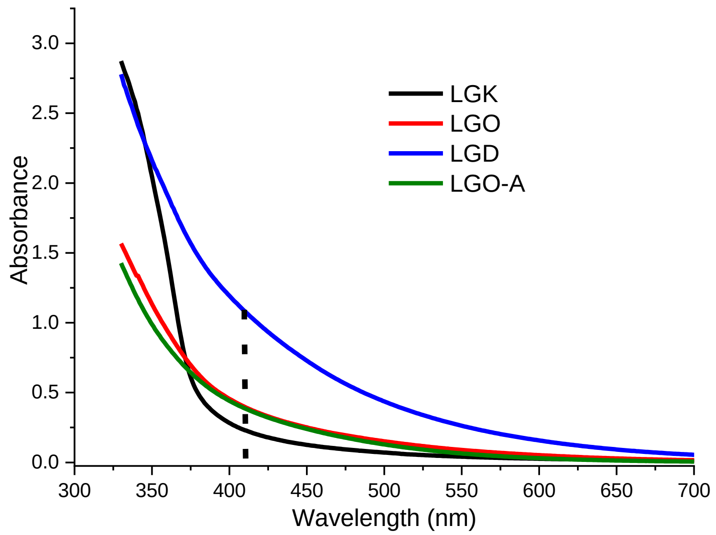

Figure S13. UV-vis absorption of lignin in THF solutions (~200 mg/L). LGD-A was not soluble.

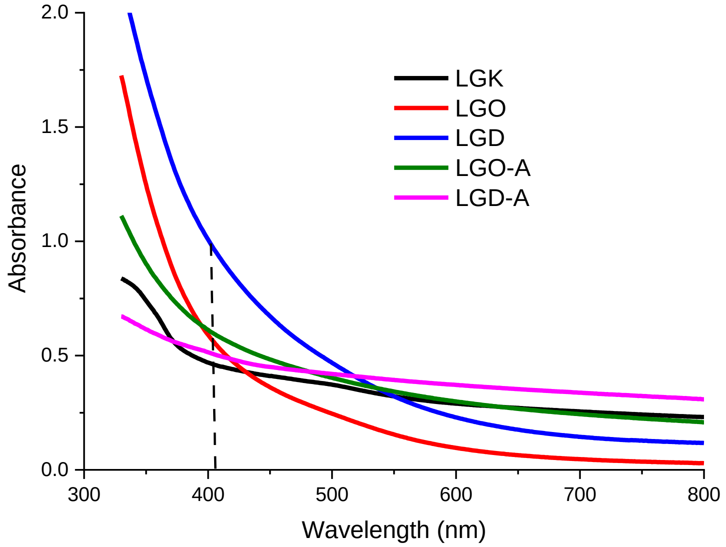

Figure S14. UV-vis absorption of lignin in the photocurable resin dispersion (~200 mg/Kg).

8. Cure depth tests

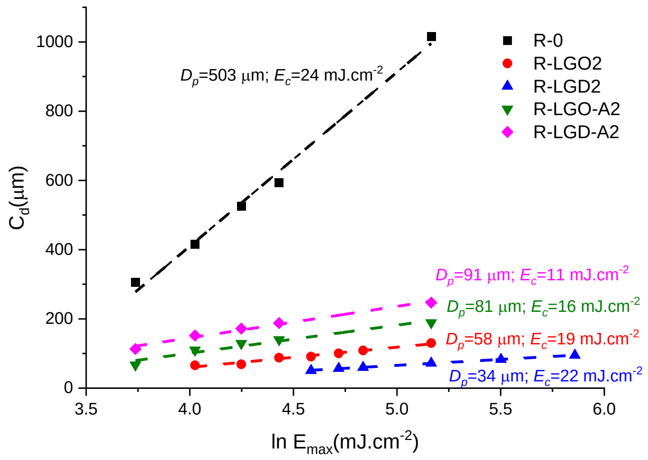

Figure S15. Jacobs working curves for the neat acrylic resin (R-0) and composites containing 2 wt.% lignin.

9. DMA of the neat acrylic resin compared with M_1 and M_2 homopolymers

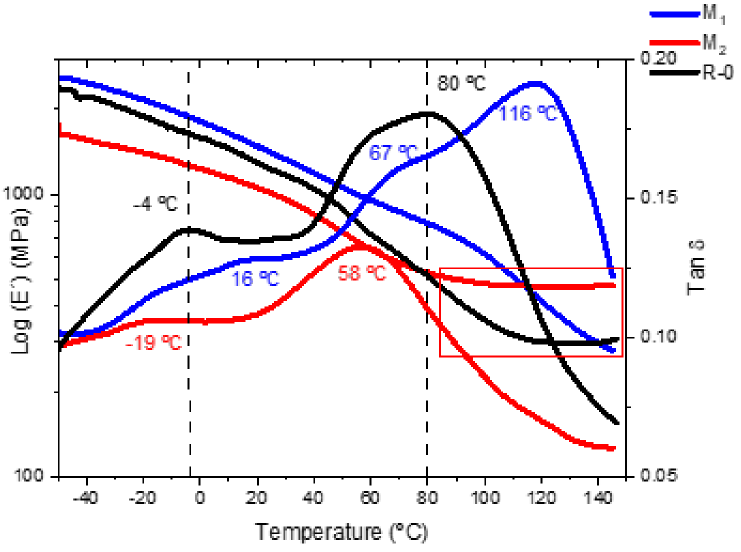

Figure S16. Logarithmic storage modulus (E′, left Y-axis) and loss tangent (tan δ, right Y-axis) as a function of temperature, measured at a frequency of 1Hz for p-M_1 (poly(PEG200DMA)), p-M_2 (poly(C10DA)) and the neat acrylic resin (R-0).

Table S1. Storage moduli at different temperatures, average molecular weight of chain segments between cross-links (M_c), cross-link density (υ_c), and glass transition temperature (T_g) as measured by DMA, along with DBC% (ATR-FTIR) and yield of insoluble fraction (YIF %) following acetone extraction.

Sample code	E′ (10^9 Pa) (T = - 48 ºC)	E′ (10^9 Pa) (T = 25 ºC)	E′ (10^9 Pa) (T = 120-140 ºC)	υ_c ($\cdot 10^{-2}$) (mol·cm^{-3})	M_c (g·mol^{-1})	T_g (ºC)	DBC% FTIR	YIF (%)
**p-M$_1$**	2.57±0.58	1.40±0.42	0.28±0.04	-	-	116±7	92.1±3.4	-
**p-M$_2$**	1.49±0.29	1.01±0.22	0.47±0.12	4.54±1.12	25.2±6.1	58±10	92.7± 0.9	-
**R-0**	2.30±0.45	1.33±0.30	0.32±0.04	3.09±0.035	37.7±4.0	80±1<br>-4±1	91.4±3.1	99.1±0.4
**R-LGO2**	2.50±0.10	1.45±0.09	0.23±0.004	2.24±0.04	51.7±0.8	65±2	92.0±0.8	99.4±0.1
**R-LGD2**	2.76±0.20	1.54±0.04	0.21±0.003	1.97±0.03	58.7±0.8	68±5	94.5±0.5	97.4±0.9
**R-LGO-A2**	2.89±0.14	1.85±0.23	1.37±0.11	3.37±0.002	35.0±0.2	78±3<br>27±6	90.4±3.0	99±1
**R-LGD-A2**	2.46±0.17	1.58±0.06	0.30±0.006	2.85±0.006	41.3±0.8	80±2<br>39±2	90.0± 3.3	100

10. Thermogravimetric analysis (TGA)

The lignin samples exhibited a three-step weight loss behavior (Figure S17). The first minor weight loss step (55-120 ºC) was attributed to the release of volatile components (<3 wt.%). The second weight loss (200 ºC- 350 °C, 10-14 wt.%), fell within the range of hemicellulose decomposition,[5] and was mainly caused by the fragmentation of the weak C─O bonds between structural units. The greatest mass loss, between 350 and 600 ºC, corresponded to lignin decomposition, including the release of aromatic rings and cleavage of C-C bonds. Beyond 600 ºC, the remaining residue (29 to 42 wt.%, depending on the lignin type) was attributed to the formation of highly condensed aromatic structures.[6,7]

As can be observed in Table S2, acrylation increased the thermal stability of organosolv lignin, increasing T_{10}, T_{onset} and ΔT_{max}, with little effect on the char residue. In contrast, LGD-A showed decreased T_{10}, T_{onset} and the residue, despite an increase in T_{max}. The reduced early-stage thermal stability is due to an increased mass loss below 250 °C, which is attributed to the formation of low-molecular-weight fragments generated during acrylation.[2]

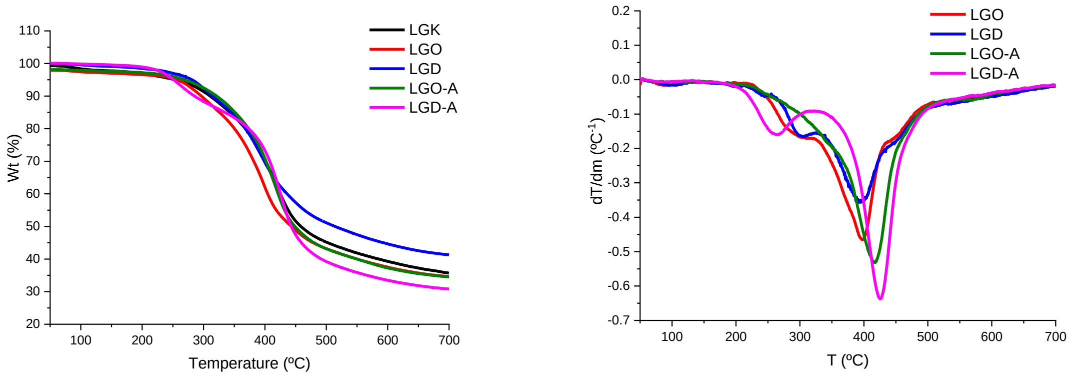

Figure S17. TGA and derivative of mass loss curves (DTG) of lignin extracts: LGO, LGD, LGO-A and LGD-A.

Table S2. Characteristic temperatures obtained from TGA thermograms. T_{10}: temperature at which 10% of the total mass is volatilized; T_{onset}: onset degradation temperature; DTG_{max}: temperature corresponding to the maximum rate of thermal decomposition; residue: non-volatile weight fraction remaining at 700 °C.

**Sample**	**T_{10} (°C)**	**T_{onset} (°C)**	**DTG_{max} (°C)**	**Residue (wt.%)**
Lignin powder				
**LGO**	316±6	258±6	302±5, 397±1	33.0±0.4
**LGD**	304±15	271±1	303±1, 395±1	42.4±0.1
**LGO-A**	322±1	341±1	355±6, 417±1	32.3±0.3
**LGD-A**	281±6	218±7	262±1, 426±1	29.2±0.4
Printed composites				
**R-0**	381±1.2	386.0.±0.9	429.0±1.0	0.5±0.2
**R-LGO2**	378.0±1.0	382.0±2.0	429.0±3.5	1.8±0.1
**R-LGO4**	371.0±1.6	381.0±0.5	427.0±0.6	2.8±.1.1
**R-LGD2**	374.0±1.5	382.0±0.7	431.5±0.8	2.3±0.4
**R-LGO-A2**	394.5±1.8	394.0±0.7	440.0±1.4	1.6±0.1
**R-LGD-A2**	395.5±1.3	395.0±2.8	443.0±1.4	1.9±0.4

The DTG curves of the neat acrylic resin and lignin composites exhibited a single degradation band between 200 and 500 ºC, which can be attributed to the cleavage of the main polymer backbone in the acrylate matrix. The neat acrylic resin left a negligible residue at 700 ºC, confirming almost complete degradation of the polymer network,[8] whereas the char residue of all the lignin composites increased proportionally with lignin content (Table S2).

11. Tensile properties and Shore D hardness

Table S3. Results of tensile tests and Shore D hardness of the neat acrylic resin and composites with varying lignin types and contents.

Formulation name	Young´s Modulus (MPa)	Stress at break (MPa)	Strain at break (%)	Tensile Toughness (kJ.m^{-2})	Shore D Hardness (ºSh)
**p-M$_1$**	653±27	45.5±1.9	15.4±1.8	103.6±9.9	-
**p--M$_2$**	398±4	25.4±0.9	9.9±0.7	30.0±3.4	-
**R-0**	406±30	31.2±1.8	13.2±1.6	48.9±6.6	78.3±1.0
**R-LGO1**	387±18	21.5±1.9	7.5±1.2	16.6±3.9	80.3±1.6
**R-LGO2**	429±13	24.1±2.0	7.5±1.1	19.9±9.5	81.1±1.3
**R-LGO3**	427±7	28.2±1.0	8.9±1.0	29.6±5.8	81.4±1.5
**R-LGO4**	443±16	29.3±0.5	9.0±1.0	30.6±7.5	81.6±1.1
**R-LGO-A2**	454±22	19.2±1.5	6.5±1.1	13.3±4.0	79.0±0.5
**R-LGD1**	400±20	19.6±2.2	6.4±0.8	13.0±3.5	80.7±0.6
**R-LGD2**	395±26	26.1±1.7	8.4±1.2	24.4±4.3	82.0±0.9
**R-LGD-A2**	422±49	19.0±2.0	5.0±1.0	8.95±3.9	78.8±0.5

12. SEM images of fractured cross-section of the neat acrylic resin and lignin composites

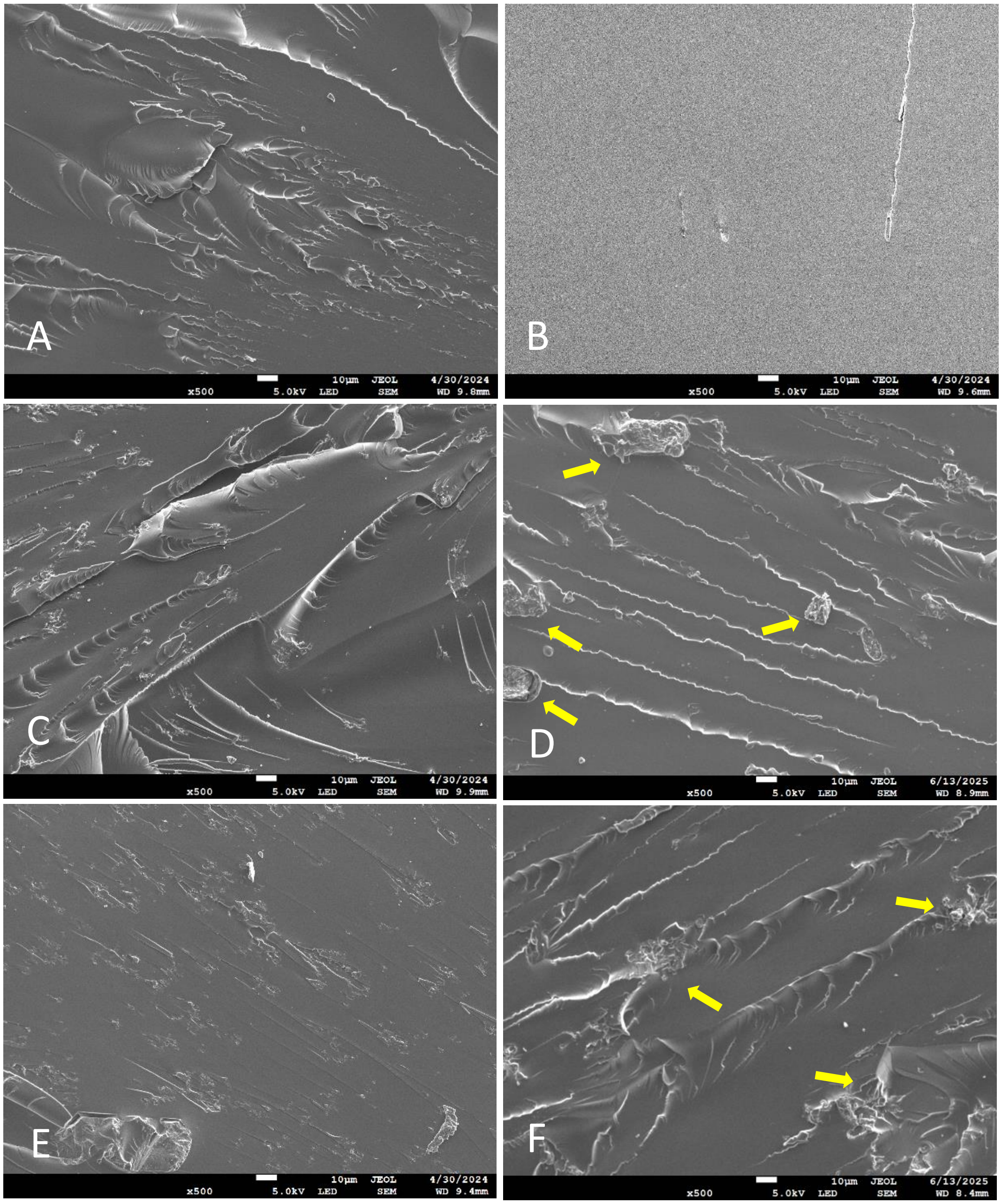

Figure S18. SEM images at 500X magnification: rough area of the neat acrylic resin (R-0) (A); smooth area of the neat acrylic resin (R-0) (B); R-LGO2 (C); R-LGO-A2 (D); R-LGD2 (E); R-LGD-A2 (F).

13. Accuracy of printed samples

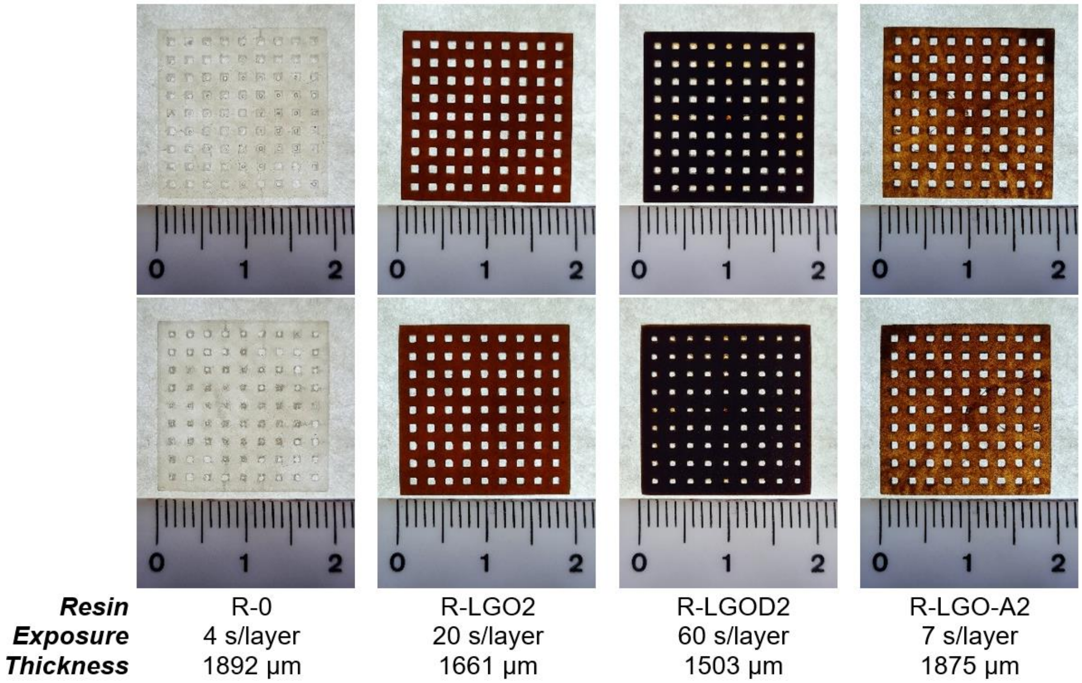

Figure S19. Images of the porous-structured square plates with square holes, printed with the neat acrylic resin and selected lignin composites, obtained with a cell-phone camera. The theoretical dimensions of the plate model were set to 19.11 x 19.11 x 2.00 mm, and the length of the square orifice side was set to 1.0 mm. First line – front sides; second line – back sides.

14. Antioxidant activity

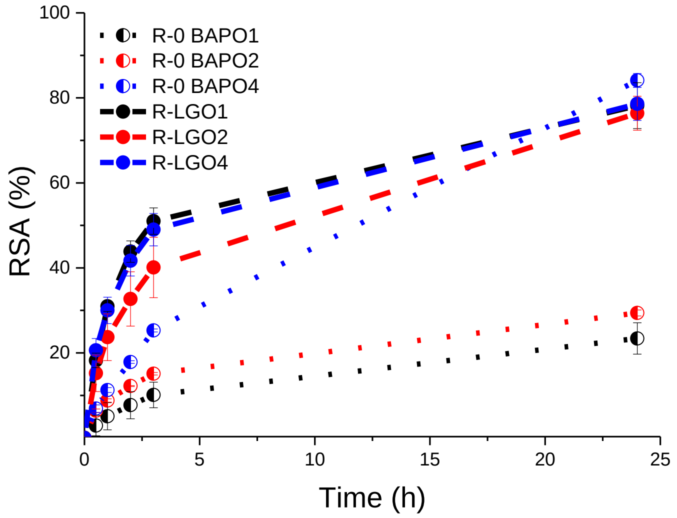

Figure S20. Radical scavenging (RSA %) of neat acrylic resin (R-0) and lignin composites: effect of photoinitiator content and increasing native organosolv lignin loading. Curves are guides to the eye and the error bars represent standard deviations.

REFERENCES

(1) Lebedevaite, M.; Talacka, V.; Ostrauskaite, J. High Biorenewable Content Acrylate Photocurable Resins for DLP 3D Printing. *J. Appl. Polym. Sci.* **2021**, *138* (16), 50233. https://doi.org/10.1002/APP.50233;WGROUP:STRING:PUBLICATION.

(2) Hua, Q.; Liu, L. Y.; Cho, M.; Karaaslan, M. A.; Zhang, H.; Kim, C. S.; Renneckar, S. Functional Lignin Building Blocks: Reactive Vinyl Esters with Acrylic Acid. *Biomacromolecules* **2023**, *24* (2), 592–603. https://doi.org/10.1021/ACS.BIOMAC.2C00806.

(3) Nevrez, L. A. M.; Casarrubias, L. B.; Celzard, A.; Fierro, V.; Mũoz, V. T.; Davila, A. C.; Lubian, J. R. T.; Snchez, G. G. Biopolymer-Based Nanocomposites: Effect of Lignin Acetylation in Cellulose Triacetate Films. *Sci. Technol. Adv. Mater.* **2011**, *12* (4), 045006. https://doi.org/10.1088/1468-6996/12/4/045006.

(4) Sutton, J. T.; Rajan, K.; Harper, D. P.; Chmely, S. C. Lignin-Containing Photoactive Resins for 3D Printing by Stereolithography. *ACS Appl. Mater. Interfaces* **2018**, *10* (42), 36456–36463. https://doi.org/10.1021/ACSAMI.8B13031.

(5) Liodakis, S.; Bakirtzis, D.; Dimitrakopoulos, A. Ignition Characteristics of Forest Species in Relation to Thermal Analysis Data. *Thermochim. Acta* **2002**, *390* (1–2), 83–91. https://doi.org/10.1016/S0040-6031(02)00077-1.

(6) Zhang, M.; Resende, F. L. P.; Moutsoglou, A.; Raynie, D. E. Pyrolysis of Lignin Extracted from Prairie Cordgrass, Aspen, and Kraft Lignin by Py-GC/MS and TGA/FTIR. *J. Anal. Appl. Pyrolysis* **2012**, *98*, 65–71. https://doi.org/10.1016/J.JAAP.2012.05.009.

(7) Wan, Z.; Zhang, H.; Niu, M.; Zhang, W.; Guo, Y.; Li, H. Preparation of Lignin Nanoparticles by Ultrasonication and Its Incorporation in DLP 3D Printing UV-Curable Resin as Bio-Filler. *Ind. Crops Prod.* **2025**, *224*, 120394. https://doi.org/10.1016/J.INDCROP.2024.120394.

(8) Goliszek, M.; Podkościelna, B.; Klepka, T.; Sevastyanova, O. Preparation, Thermal, and Mechanical Characterization of UV-Cured Polymer Biocomposites with Lignin. *Polymers 2020, Vol. 12,* **2020**, *12* (5). https://doi.org/10.3390/POLYM12051159.
